# Sweepstake reproductive success and collective dispersal produce chaotic genetic patchiness in a broadcast spawner

**DOI:** 10.1126/sciadv.abj4713

**Published:** 2021-09-10

**Authors:** David L. J. Vendrami, Lloyd S. Peck, Melody S. Clark, Bjarki Eldon, Michael Meredith, Joseph I. Hoffman

**Affiliations:** 1 Department of Animal Behaviour, Bielefeld University, Postfach 100131, 33501 Bielefeld, Germany.; 2 British Antarctic Survey, High Cross, Madingley Road, Cambridge CB3 OET, UK.; 3 Leibniz Institute for Evolution and Biodiversity Research, Museum für Naturkunde, 10115 Berlin, Germany.

## Abstract

A long-standing paradox of marine populations is chaotic genetic patchiness (CGP), temporally unstable patterns of genetic differentiation that occur below the geographic scale of effective dispersal. Several mechanisms are hypothesized to explain CGP including natural selection, spatiotemporal fluctuations in larval source populations, self-recruitment, and sweepstake reproduction. Discriminating among them is extremely difficult but is fundamental to understanding how marine organisms reproduce and disperse. Here, we report a notable example of CGP in the Antarctic limpet, an unusually tractable system where multiple confounding explanations can be discounted. Using population genomics, temporally replicated sampling, surface drifters, and forward genetic simulations, we show that CGP likely arises from an extreme sweepstake event together with collective larval dispersal, while selection appears to be unimportant. Our results illustrate the importance of neutral demographic forces in natural populations and have important implications for understanding the recruitment dynamics, population connectivity, local adaptation, and resilience of marine populations.

## Teaser

Extreme variation in reproductive success and correlated larval dispersal produce chaotic genetic patchiness in a marine mollusc.

## INTRODUCTION

Knowledge of the mechanisms shaping the genetic diversity of marine organisms is essential for understanding metapopulation and community dynamics (*1*, *2*), for predicting future responses to anthropogenic challenges (*3*), for marine protected area design and fisheries management (*4*, *5*), and for the development of ecological and evolutionary theory (*6*, *7*). However, it has proven challenging to reconcile the results of population genetic studies with theoretical expectations. In particular, many marine species with dispersive larvae have orders of magnitude smaller effective population sizes (*N*_e_) than would be expected from their census sizes, carry unexpectedly high genetic loads, and are genetically structured over space and/or time (*8*–*10*). These paradoxical observations challenge the paradigm that marine populations are open and well connected and suggest that recruitment dynamics may be more complex than previously anticipated (*6*).

A prime example of the disconnect between empiricism and theory is chaotic genetic patchiness (CGP). This term was coined by Johnson and Black (*11*) to describe unexpected patterns of genetic differentiation that occur over fine geographic scales and which are unstable over time. These patterns are not expected according to classical population genetic theory because they occur below the effective range of larval dispersal, meaning that genetic structuring should be counteracted by gene flow (*7*). Moreover, the strength of genetic differentiation observed over fine geographic scales can be of similar magnitude to the strength of genetic differentiation observed over scales of hundreds to thousands of kilometers (*12*). This suggests that the mechanisms responsible for CGP should be capable of generating fine-scale genetic structure while at the same time not increasing the strength of differentiation over large geographic scales (*13*).

CGP raises a number of important questions about the microevolutionary forces at play in marine populations and emphasizes the need for studies of the precise mechanisms generating fine-scale patterns of genetic divergence (*14*). Far from being routine descriptions of genetic variation, these studies are essential for understanding the life histories, reproductive ecologies, population dynamics, and evolutionary genetics of marine species (*7*). They are also important from an applied perspective when genetic data are used to inform practical conservation and marine policy (*10*).

Several mechanisms have been hypothesized to explain CGP. First, environmental heterogeneity may drive selection for locally beneficial alleles through the differential survival of recruits (*11*). Second, temporal changes in ocean currents may cause successive waves of recruits to originate from genetically distinct source populations [the “variable sources hypothesis” (*15*, *16*)]. Third, fine-scale genetic differences could accumulate via self-recruitment, which occurs when larvae settle in their source population (*17*, *18*). Fourth, according to the “sweepstakes reproductive success” hypothesis, reproduction in marine species with high fecundity and poor early survival is a lottery with a handful of winners that succeed by chance in matching their reproductive activity to oceanographic conditions conducive to spawning, fertilization, larval development, and recruitment (*8*). This results in strong local genetic drift during the larval stage and a subsequent reduction in the genetic diversity of recruits. A final mechanism is collective dispersal, whereby groups of larvae from the same location do not diffuse at random but instead show correlated dispersal pathways (*14*, *19*). Collective dispersal counteracts the homogenizing influence of gene flow but may not be sufficient on its own to explain CGP (*7*, *10*, *14*).

While all of these hypotheses generate specific predictions, in practice, empirical studies have struggled to separate the effects of different mechanisms (*7*, *10*, *15*, *20*). For example, the hypothesis of selection was initially supported by reports of associations between allozymes and environmental variables over fine geographic scales (*21*, *22*). However, subsequent studies have uncovered consistent patterns across multiple presumed selectively neutral loci, which are difficult to reconcile with direct selection (*10*). Nevertheless, too few genetic markers have so far been used to rule out the possibility of selection acting at linked loci, which is amenable to investigation using a genome scan approach (*23*).

Marine systems are also highly complex, which usually precludes teasing apart different mechanisms in natural settings. In particular, the multiple sources hypothesis often cannot be discounted due to the presence of large-scale population genetic structure (*15*, *24*). Moreover, sweepstakes reproductive success should leave distinct imprints on larval cohorts including reduced genetic diversity, lower *N*_e_, and, in extreme cases, the presence of close kin (*8*). However, the same patterns can be produced by self-recruitment (*17*, *18*, *20*), which appears to be more common in marine populations than was previously assumed (*18*, *25*).

An additional challenge is that systematic temporal replication is necessary to confirm a fundamental tenet of CGP, temporal instability, as well as to distinguish ephemeral signatures associated with sweepstake reproductive success (*8*) from potentially more stable patterns that may arise from self-recruitment (*17*, *26*). Moreover, temporal samples may need to be separated by as many as three to five generations to confirm or falsify temporal instability in species with overlapping generations (*8*, *27*). This standard can rarely, if ever, be achieved given the generation times of many marine organisms and the short duration of a typical research grant (*8*).

Another issue is statistical power, which can result in the failure to detect genuine sweepstake signatures, especially when small numbers of genetic markers are used (*8*). Fortunately, population genomic approaches such as restriction site–associated DNA (RAD) sequencing (*28*) allow the genotyping of tens to hundreds of thousands of single-nucleotide polymorphisms (SNPs). These approaches offer far greater power to characterize fine-scale genetic structure (*29*), to interrogate patterns of kinship (*30*), and to test for patterns of selection across the genome (*23*).

Last, empirical studies of CGP rarely, if ever, substantiate their conclusions with quantitative estimates of what should have been observed in theory (*7*). Two approaches may be useful in this regard. First, forward genetic simulations (*31*) allow realistic evolutionary scenarios to be modeled to predict their impacts on genomic data. These versatile models can incorporate population size changes, patterns of migration, and complex mating schemes, from assortative mating to sequential mate choice. In this way, observed patterns in genomic data can be connected to key underlying processes such as reproduction and migration.

Second, sweepstake reproduction in a highly fecund population occurs when a small number of diploid parents contribute large numbers of offspring (in mathematical terms on the order of the population size) to the next generation. When one traces ancestral lineages of a sample of gene copies back in time, many of these lineages will merge at the time of a sweepstakes reproduction event, because multiple ancestral lineages can be involved in the event (*32*–*34*). This is in contrast to the Kingman coalescent, where, at most, two ancestral lineages can merge at any given time (*35*–*37*). This is because, in low-fecundity populations where only small families (in comparison to the total population size) are observed, the probability of more than two ancestral lineages being involved in any given reproduction event becomes negligible in a large population (*38*). Consequently, it should be possible to test for genomic footprints of sweepstake reproduction by asking whether allele frequency spectra predicted by a multiple-merger coalescent show greater similarity to empirical allele frequency spectra than those predicted by the Kingman coalescent (*7*, *39*, *40*).

An outstanding opportunity to investigate the mechanisms responsible for CGP is provided by the Antarctic limpet, *Nacella concinna*. This widespread and highly abundant shallow-water macroinvertebrate is a classical broadcast spawner with free-swimming planktonic larvae that persist in the water column for around 40 to 45 days depending on the temperature (*41*, *42*). The eggs are free-spawned into the water column, are negatively buoyant, and sink to the seabed. After fertilization, the embryos develop inside the egg membrane for the first 75 hours before hatching into free-swimming gastrulae (*41*). Shortly afterward, these metamorphose into trochophore larvae, which swim very actively and aggregate toward the surface (*41*, *43*), implying that larval transport likely occurs in the uppermost layers of the ocean. In line with this, Bowden *et al.* (*43*) collected larvae throughout the spawning period of *N. concinna* with a plankton net near the surface and correlated the disappearance of the larvae with the arrival of very small juveniles on settlement panels and shallow rocks.

**Fig. 1.**
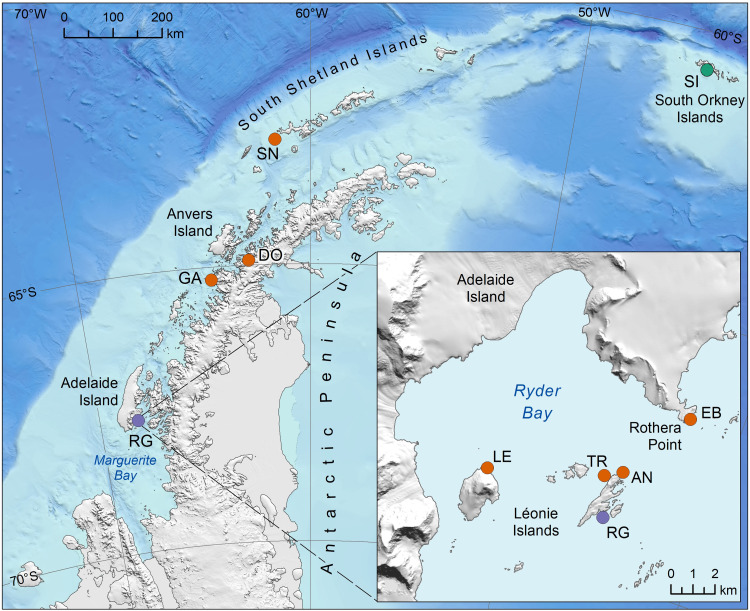
Map of *N. concinna* sampling sites. The main figure shows the macrogeographic-scale sites, which were sampled in 1999. The inset shows the microgeographic-scale sites, which were sampled in both 1999 and 2015. The sampling locations are color-coded according to their membership to one of three inferred genetic groups (see Results for details). Macrogeographic scale: SI, Signy Island; SN, Snow Island; DO, Dobrowolski Island; GA, Galindez Island; RG, Rose Garden; microgeographic scale: TR, Trolval; AN, Anchorage North; LE, Leonie North East; EB, East Beach. RG 1999, depicted in purple, was common to both geographic scales. Data sources: Bathymetry (*78*), land terrain (*79*), and coastlines (*80*). Figure produced by L. Gerrish, British Antarctic Survey.

*N. concinna* can be readily sampled along the Antarctic Peninsula, the most proximate projection of the Antarctic land mass to another continent and an area that is ideally suited to investigating the effects of geographical isolation and oceanography on genetic connectivity (*44*). Previous studies using amplified fragment length polymorphisms (AFLPs) have shown that this species is largely unstructured along the Peninsula, apart from genetic differences involving Signy Island, which lies to the north of the Peninsula in the South Orkney Islands, and Rose Garden, which is situated at the southern edge of accessible coastline in Ryder Bay, Adelaide Island (*45*). By contrast, fine-scale genetic differentiation has been observed within Ryder Bay over a scale of just a few kilometers (*46*), mainly involving Rose Garden and nearby sites around Anchorage Island (Fig. 1). *N. concinna* therefore exhibits clear hallmarks of CGP and may even represent an idealized example given the large geographic expanse over which population structure is absent.

Here, we investigated the mechanism(s) responsible for CGP by combining population genomics with a hierarchical sampling design and temporally replicated fine-scale sampling within Ryder Bay, separated by 16 years or approximately three *N. concinna* generations. Our use of population genomics substantially increases the power to detect fine-scale genetic differentiation and to test for signatures of CGP associated with different mechanisms, including natural selection. Furthermore, the absence of large-scale population structure in *N. concinna* allows us to effectively rule out the multiple sources hypothesis, while self-recruitment also appears unlikely given the extended obligate larval phase of *N. concinna* in combination with local circulation patterns, which we have inferred from surface drifters deployed in Ryder Bay.

We show that a single cohort sampled from Rose Garden in 1999 deviates from an overall pattern of approximate spatial and temporal panmixia and exhibits multiple signatures of an extreme sweepstake event including reduced genetic diversity, locally elevated linkage disequilibrium (LD), and the presence of multiple full- and half-siblings. These signatures can be reproduced by neutral simulations invoking strong genetic drift and collective dispersal, while temporally replicated genome scans do not reveal any evidence for a role of selection. Last, all of the cohorts show a better fit to a multiple-merger than to a Kingman coalescent, indicating a species-wide effect of sweepstakes reproduction. Our study provides compelling evidence for a dominant role of local genetic drift in producing CGP, with far-reaching implications for understanding recruitment dynamics, population connectivity, local adaptation, and the resilience of marine populations.

## RESULTS

### Genomic data

Sampling cohorts were pooled at random within three RAD sequencing libraries (table S1) and sequenced on three Illumina X Ten lanes within the same flow cell. The resulting 575,970,000 cleaned and demultiplexed 150–base pair (bp) paired-end Illumina sequence reads were de novo assembled into 150,434 RAD loci and used to call a total of 3,991,653 raw SNPs. Application of the stringent filtering criteria described in Supplementary Methods resulted in a final dataset consisting of 134 *N. concinna* individuals from 14 cohorts genotyped at 109,760 SNPs. Data from the three sequencing libraries were comparable, with phred-scaled quality scores being uniformly high and the average depth of coverage of the SNPs retained in our final dataset showing no substantial differences among the libraries [analysis of variance (ANOVA), *F* = 1.5, *P* = 0.23; table S1].

### Genetic structure and CGP

**Fig. 2.**
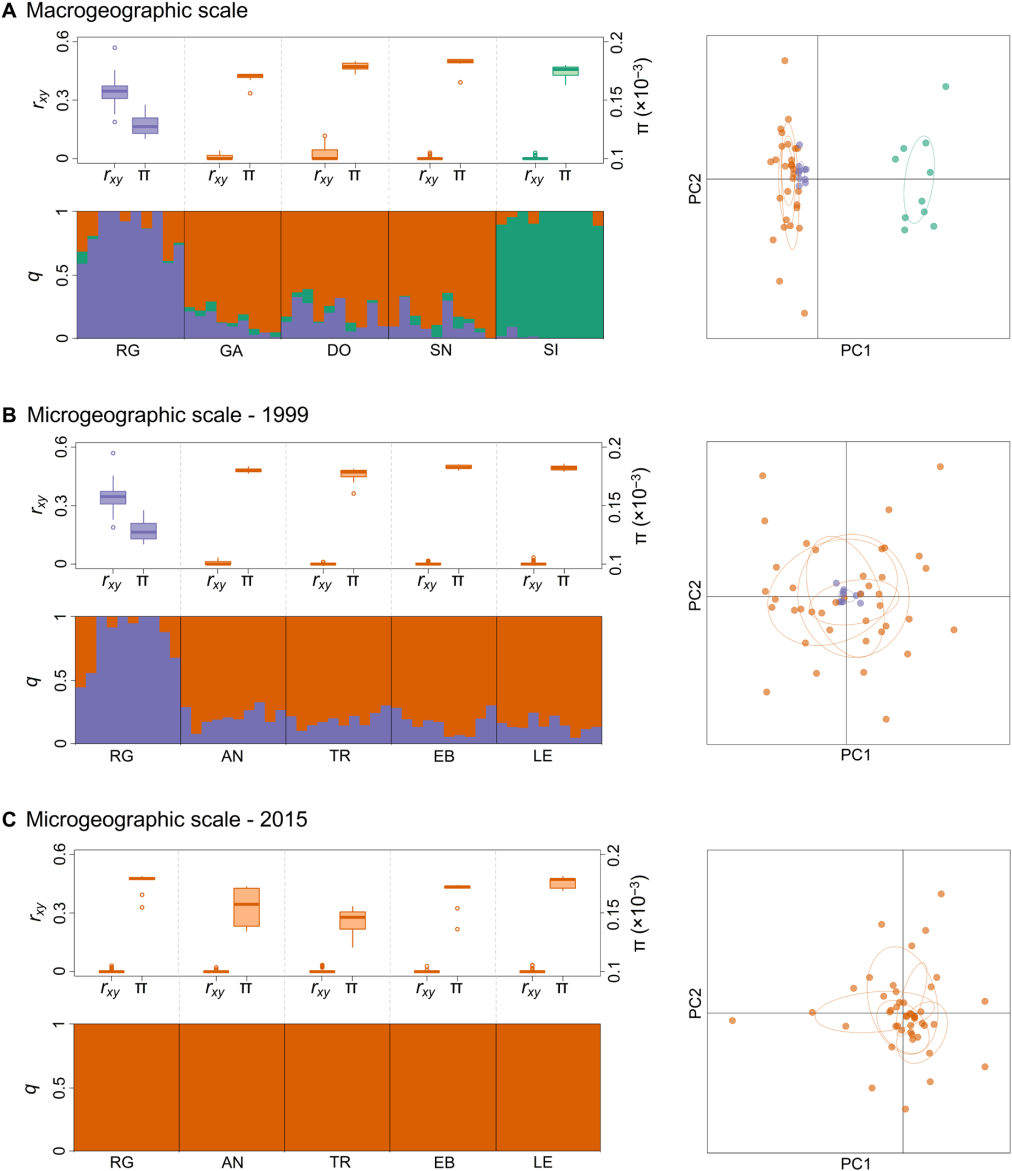
Spatiotemporal patterns of genetic structure in *N. concinna*. Results are shown separately for (**A**) the macrogeographic scale in 1999, (**B**) the microgeographic scale in 1999, and (**C**) the microgeographic scale in 2015. Tukey boxplots show variation within and among cohorts in pairwise genomic relatedness (*r_xy_*) and nucleotide diversity (π). Barplots show the results of the sparse nonnegative matrix factorization (sNMF) analysis, where each vertical bar corresponds to an individual whose group membership coefficient (*q*) has been partitioned into different colors according to the posterior probability of membership to each of three inferred genetic groups. Scatterplots depict individual variation in principal component (PC) scores derived from PCA of the genomic data. All of the panels have been color-coded according to the results of the sNMF analysis.

We used three complementary approaches—pairwise *F*_st_ comparisons, a Bayesian clustering approach, and principal components analysis (PCA)—to resolve population genetic structure separately for each geographic scale and time point. In line with previous AFLP studies (*45*, *46*), we found evidence for population genetic structure on the macrogeographic scale involving Signy in the South Orkney Islands and, to a lesser extent, Rose Garden (Fig. 2A and table S2). On the microgeographic scale, all of the sampling cohorts were genetically undifferentiated apart from Rose Garden in 1999 (Fig. 2, B and C, and table S2). This cohort occupies a central position in the PCA plot shown in Fig. 2B but shows very little scatter, indicating that its main distinguishing feature is lower genetic diversity. Analysis of the full dataset combining both geographic scales and time points revealed little in the way of structure apart from genetic differences involving Signy and Rose Garden in 1999 (table S3). Hence, *N. concinna* exhibits a transient pattern of fine-scale population structure against a backdrop of large-scale spatial and temporal genetic homogeneity, in accordance with established definition of CGP.

### Drifter data

**Fig. 3.**
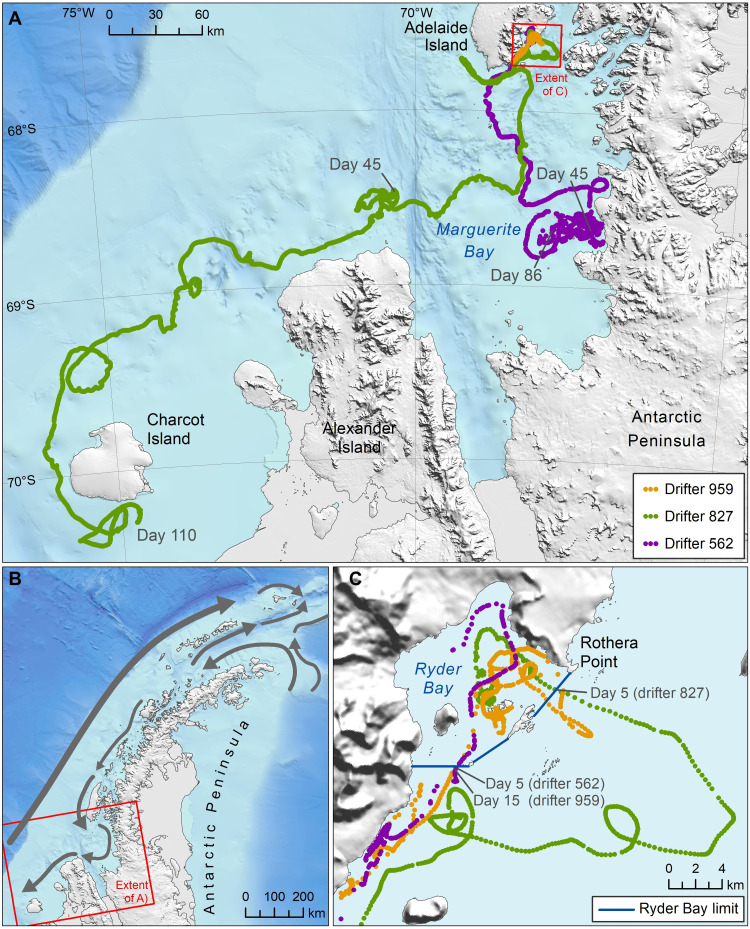
Patterns of current circulation in and around Ryder Bay. (**A**) Example traces of three drifters deployed in Ryder Bay. Points represent consecutive GPS locations, color-coded by drifter as shown in the legend. The positions of the drifters on day 45, corresponding to the approximate larval duration of *N. concinna*, and the end of each deployment are shown. (**B**) Schematic of the main currents along the Antarctic Peninsula and around Adelaide Island [based on data from (*81*–*83*)], with arrow thickness being proportional to the strength of flow. (**C**) Close-up of Ryder Bay showing the time that it took each drifter to leave the bay (i.e., to pass the boating limit, shown in blue). Data sources: Bathymetry (*78*), land terrain (*79*), and coastlines (*80*). Figure produced by L. Gerrish, British Antarctic Survey.

To investigate the potential for self-recruitment, we asked whether larvae released into Ryder Bay are likely to be retained for long enough to settle within a few meters of their point of origin around 40 to 45 days later. To shed light on local circulation patterns, we deployed three surface drifters within the bay during the spawning period of *N. concinna* in 2012 (see Materials and Methods for details). To focus data collection within Ryder Bay, attempts were made to recover and redeploy the drifters whenever they approached the boating limit, which demarcates the edge of the bay. Drifter circulation patterns (representative traces are shown in Fig. 3, A and C) confirmed concepts of general circulation within and around Ryder Bay (Fig. 3B), which depict significant wind-forced variability superposed on a generally cyclonic circulation, with inflow to the bay on the southern side adjacent to Anchorage and Lagoon Islands and outflow along the northeastern side. The drifter data were also consistent with observations of iceberg movements in the bay and diver observations of moderately strong currents around the outside of Anchorage Island and at Rose Garden. None of the drifters remained within 100 m of their deployment site or subsequently returned to within 100 m of their deployment site, and all of the drifters approached the boating limit within a few days of being deployed (range = 5 to 15 days). In the final deployments, which were not intercepted at the boating limit, the drifters travelled distances of tens to hundreds of kilometers toward the south of Adelaide Island (Fig. 3A).

### Genome scans

We implemented temporally replicated genome scans on the microgeographic scale to test for footprints of selection. Our rationale was that, if natural selection drives CGP, outlier loci should be identified in 1999 where CGP was present but not in 2015 where CGP was absent. Temporal replication should furthermore guard against spurious signals reflecting shared demographic histories, as these would be expected to influence both time points more or less equally. We specifically used a Bayesian hierarchical modeling approach implemented in BayPass (*47*) to test for signatures of divergent selection while accounting for the underlying genetic structure of the dataset (see Supplementary Methods for details). No loci exhibiting genome-wide significant patterns of genetic divergence were identified at either time point, regardless of whether the data were filtered for Hardy-Weinberg equilibrium (fig. S1, A and B) or not (fig. S1, C and D). This suggests that selection is unlikely to have played an appreciable role in generating the observed pattern of CGP in *N. concinna*.

### Genomic signatures of a sweepstake event

We tested a number of specific predictions of the sweepstake reproductive success hypothesis. First, we quantified each cohort’s genome-wide diversity (as nucleotide diversity, π) and calculated genomic estimates of pairwise relatedness (PI_HAT, indicated as *r_xy_* in Fig. 2). The Rose Garden 1999 cohort showed clear signatures of a sweepstake event including substantially elevated genomic relatedness and reduced π (Fig. 2, A and B). The other cohorts had pairwise genomic relatedness values centered around zero and consistently higher values of π, apart from Anchorage North and Trolval in 2015 where π was somewhat lower (Fig. 2C).

**Fig. 4.**
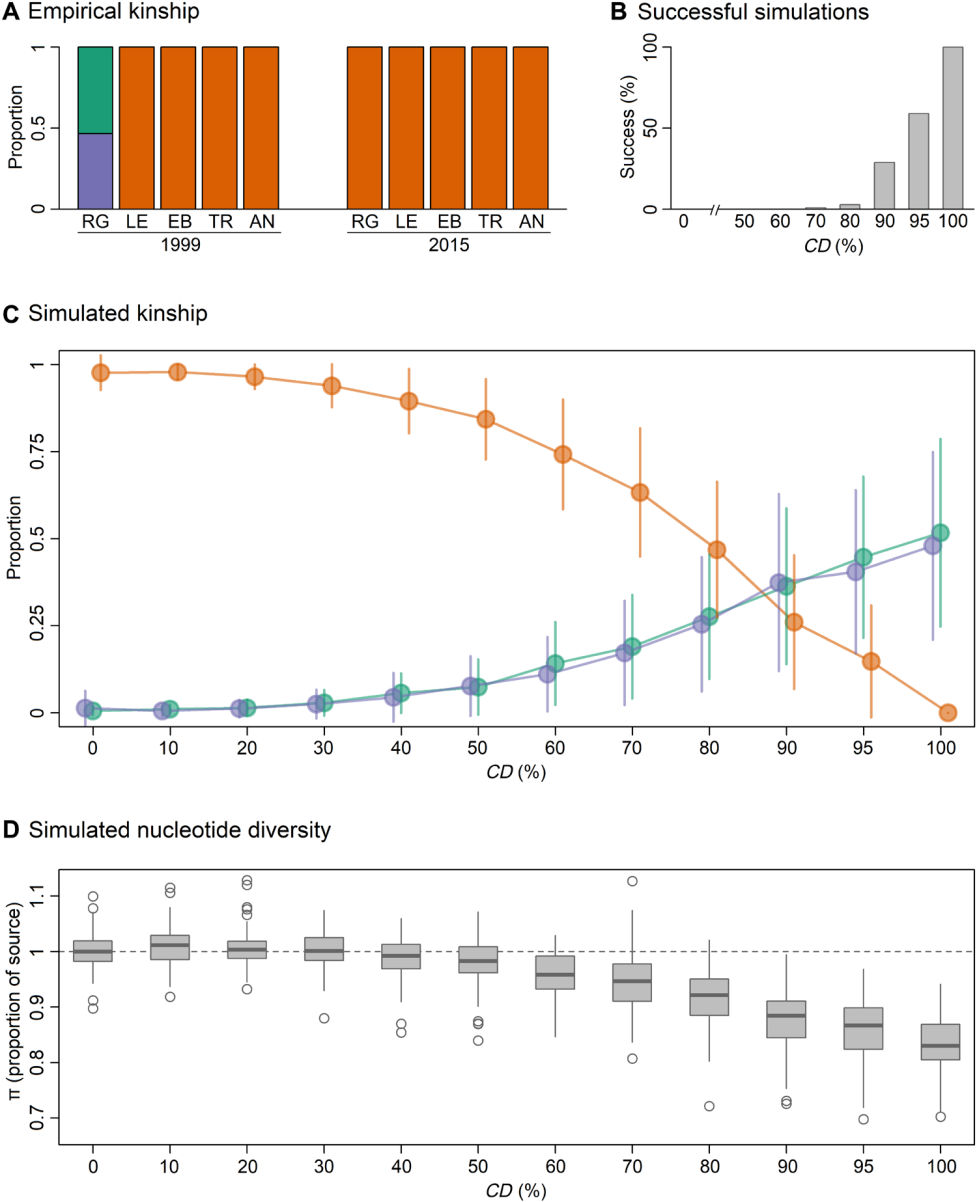
Results of forward genetic simulations. (**A**) Empirical proportions of unrelated individuals (orange), half-siblings (green), and full-siblings (purple) in *N. concinna* cohorts sampled on the microgeographic scale. Full names of the sampling sites are given in the legend of Fig. 1. (**B** to **D**) Results of sweepstake simulations. We simulated a total of five populations including a source and a sink population and allowed varying proportions of the offspring generated at the source population to collectively disperse to the sink (*CD*), while all other migration among populations occurred according to a stepping stone model (see Supplementary Methods for details). We explored the effects of varying *CD* on the kinship structure of the resulting offspring cohort at the sink population, which was subsampled to 10 individuals to reflect our empirical sample size. A simulation was regarded as having successfully reproduced our empirical results for Rose Garden 1999 when the resulting offspring sample contained only full- and half-siblings. (B) Proportion of successful simulations with increasing *CD*. (C) Summary of the kinship structure of the subsampled simulated offspring cohorts. The proportion of unrelated individuals, full-siblings, and half-siblings are shown in orange, purple, and green, respectively, with points and vertical bars representing the mean and SDs of 100 simulations, respectively. (D) Reduction in the nucleotide diversity (π) of the sink population relative to the source population for increasing values of *CD*.

To investigate further, we used the genomic data to assign pairs of individuals within cohorts to specific kinship categories following the approach of Manichaikul *et al.* (*30*). This is based on the relatedness coefficients *Z*0, *Z*1, and *Z*2, which reflect the proportion of the genome where a pair of individuals shares zero, one, or two alleles identical by descent (IBD). In accordance with the expectation that an extreme sweepstake event should result in the presence of close kin within sampled cohorts (*8*), we found that Rose Garden in 1999 was exclusively represented by full- and half-siblings, whereas all of the other cohorts comprised unrelated or very distantly related individuals (fifth-degree relatives or higher; Fig. 4A). Virtually identical results were obtained with KING-robust kinship values, which were strongly correlated with PI_HAT (*r* = 0.98, *P* < 0.01) and confirmed the absence of close relatives from all of the sampling cohorts except for Rose Garden in 1999. In support of the previous results, this approach also assigned full- or half-sibling status to 43 of 45 pairs of individuals within Rose Garden, while the remaining two pairwise comparisons were assigned as borderline third-degree relatives.

Sweepstake events should also increase LD and reduce *N*_e_ (*48*, *49*). We therefore quantified pairwise LD values across all loci and estimated *N*_e_ for all of the sampled cohorts using the LD and molecular coancestry approaches implemented in *N*_e_ estimator (*50*). The highest pairwise LD estimates were obtained for Rose Garden in 1999, Trolval in 2015, and Anchorage North in 2015 (table S4). The LD method produced *N*_e_ estimates in the order of 150 to 300 for most of the cohorts but failed to converge for Rose Garden 1999 and Trolval 2015 (table S5). The molecular coancestry model yielded substantially lower *N*_e_ estimates, reflecting a known downward bias in the method (*50*), but estimates were outputted for all of the cohorts, and these were around two to four times smaller for Rose Garden in 1999 and Anchorage North in 2015 (table S5).

### Forward genetic simulations

To investigate the strength of sweepstake reproductive success needed to replicate the main features of our empirical results, we implemented forward genetic simulations as described in Supplementary Methods. First, we investigated the conditions capable of reproducing the pattern of kinship observed at Rose Garden in 1999. Because *N. concinna* breeds in spawning aggregations or “stacks” of up to 20 individuals comprising roughly equal numbers of males and females (*51*), at the start of each generation we randomly built stacks of up to 20 individuals with a 1:1 sex ratio. We then selected a focal stack at random and allowed individuals within that stack to randomly mate until the carrying capacity (*K*) of the population was reached. We explored the effects of varying the number of reproducing females within the stack (*F*), the size of the stack (*St*), and *K* on the kinship structure of the resulting offspring cohort, which was subsampled to 10 individuals to reflect our empirical sample size. A simulation was regarded as having successfully reproduced our empirical results when the resulting offspring sample contained only full- and half-siblings. Overall, scenarios involving a single reproducing female provided by far the best fit to the empirical data (fig. S2), while the optimal combination of *K* and *St* was 1000 and 10, respectively. By contrast, the number of simulations that successfully reproduced our empirical results was negligible when females from more than one stack were allowed to contribute offspring to the next generation (fig. S3). By implication, the Rose Garden 1999 cohort most likely originated from a single female residing within one stack.

Next, we investigated the importance of collective dispersal for detecting sweepstake signatures. We simulated a generalized scenario involving five populations including a “source” and a “sink” population. Reproduction in the source population followed the sweepstake model described above, with *F* = 1, *K* = 1000, and *St* = 10. Reproduction in the other four populations was less strict, with multiple stacks producing offspring and multiple females breeding within stacks. We allowed varying proportions of the offspring generated at the source population to collectively disperse (*CD*) to the sink population, while all other migration among populations occurred following a stepping stone model until carrying capacities were reached. We found that, while a small proportion of simulations with *CD* as low as 70% were capable of reproducing the kinship structure of Rose Garden in 1999, only simulations with *CD* greater than or equal to 95% resulted in a decline in π comparable to the difference in π between Rose Garden in 1999 and 2015 (Fig. 4).

In our simulations, declines in π were always accompanied by the occurrence of close kin within cohorts. Consequently, it is unlikely that a sweepstake event could have directly reduced π without leaving a signature of increased relatedness in the sampled cohorts from Anchorage North and Trolval in 2015 (Fig. 2C). We therefore allowed the previous simulations to run for a further three generations without enforcing any additional sweepstake events. One generation after the sweepstake event, stepping stone migration resulted in slightly reduced π and a small proportion of close kin in simulated cohorts from populations immediately adjacent to the sink (fig. S4A). Two generations after the sweepstake event, simulated cohorts from the sink population exhibited reduced π but close kin were no longer present (fig. S4B). Three generations after the sweepstake event, a similar pattern was again observed, but this time in the populations adjacent to the sink (fig. S4C). These patterns arise because outcrossing produces unrelated offspring, yet low-diversity haplotypes from the original sweepstake event continue to segregate in these populations, reducing π. Consequently, our results suggest either that independent sweepstake events occurred at Anchorage North and Trolval around two generations before sampling in 2015 or that these cohorts carry signatures of sweepstake events that occurred at other localities in previous generations.

### Effect of sweepstakes reproduction on allele frequency spectra

The genealogical trees of samples taken from populations with sweepstakes reproduction are characterized by multiple mergers of ancestral lineages (*32*–*34*). We therefore compared population-specific empirical allele frequency spectra with those simulated under two models: a multiple-merger Xi-Beta(2-α, α), with 1 < α < 2, coalescent obtained from a population model of high fecundity and sweepstakes reproduction (*34*, *52*), and the Kingman-coalescent, which is appropriate for describing genetic variation in low-fecundity populations (*35*–*37*). To evaluate the fit of the empirical data to each model, we calculated the *l*_2_ distance, which is a natural measure of the deviation of the predictions of the model from the data points, with smaller values indicating a better fit. Table S6 shows comparisons of observed and expected site frequency spectra using the *l*_2_ metric for both coalescent models. All of the cohorts showed a better fit to the Xi-Beta(2-α, α) coalescent. We interpret this as indicating that sweepstakes reproduction is not restricted to Rose Garden, Anchorage North, and Trolval but is a characteristic of the species as a whole.

## DISCUSSION

Understanding CGP remains a key challenge in marine ecology owing to the difficulty of distinguishing among alternative hypothesized mechanisms in the wild (*7*, *10*, *15*, *20*). We therefore combined population genomics with temporal sampling to investigate the drivers of CGP in an Antarctic broadcast spawner with long-lived dispersive larvae. We uncovered a notable empirical example of CGP that is best explained by an extreme sweepstake effect together with collective dispersal. By effectively ruling out a number of confounding mechanisms, our study lends weight to the argument that CGP can be generated by strong local genetic drift without the need to invoke selection (*8*, *14*).

Our focus on *N. concinna* was motivated by previous AFLP studies reporting fine-scale genetic structure within Ryder Bay against a backdrop of large-scale panmixia along the Antarctic Peninsula (*45*, *46*). We have now confirmed this pattern using genomic data while also adding a temporal component that supports a key tenet of CGP, temporal instability (*8*). The fact that *N. concinna* is largely unstructured over both space and time argues against the multiple sources hypothesis, especially because Signy Island bears no resemblance to the Rose Garden 1999 sampling cohort, implying that long-distance larval transport from a population to the north of the Antarctic Peninsula is unlikely to explain our results.

Self-recruitment also appears to be unlikely given that Antarctic limpet larvae persist in the water column for around 40 to 45 days (*41*). Furthermore, early larval behavior involves a phase of swimming up into the water column for several hours to days, which, combined with water movements, would exclude larvae from being held close to the spawning locality (*41*). This should effectively preclude self-recruitment, especially at Rose Garden, which experiences perceptible current flows due to its exposed location on the outer edge of Ryder Bay. To investigate further, we deployed surface drifters in the bay to shed light on general circulation patterns. The resulting tracks indicated that the likelihood of a given particle returning to a location already occupied 40 to 45 days later was very small. Specifically, none of the drifter deployments remained within Ryder Bay for more than around 2 weeks, while the final uninterrupted deployments revealed long southward journeys. Although these results are only strictly relevant to the times that the drifters were deployed and to the locations that they occupied, they lend further support to the argument that self-recruitment is unlikely to explain CGP in *N. concinna*.

By contrast, we identified a suite of correlated genomic signatures associated with an extreme sweepstake event, including locally reduced genetic diversity, elevated LD, and decreased *N*_e_. Notably, for a broadcast spawner with long-lived planktonic larvae, the Rose Garden 1999 sampling cohort was composed exclusively of full- and half-siblings. This is consistent with previous studies reporting similar, albeit far less extreme, patterns of relatedness (*16*, *20*, *53*) and kinship (*15*, *17*, *26*, *54*) in marine populations. However, the precise mechanisms responsible for these patterns have remained open to question, mainly due to the difficulty of disentangling sweepstake reproductive success from self-recruitment (*17*, *53*, *54*). Our findings are therefore important because they show that sweepstake effects are capable of generating spatially clustered family groups in a natural system where self-recruitment is unlikely to be important.

To further substantiate our conclusions, we used forward genetic simulations to evaluate the likely conditions that could give rise to our empirical results. As anticipated, simulations involving a single reproducing female residing in a discrete stack were 100% successful at replicating the observed kinship structure of Rose Garden in 1999, whereas simulations involving two or more females rarely produced samples comprising only close kin. Furthermore, simulations involving multiple reproducing stacks also had a negligible success rate. This suggests that the sweepstake event responsible for producing the Rose Garden 1999 cohort must have been extreme and probably involved one or, at most, two reproducing females. Our empirical results and simulations therefore support the notion that, although many marine organisms have very large census population sizes, on a local scale breeding groups can be tiny, resulting in strong genetic drift.

A further insight from our simulations is that CGP is unlikely to arise in the absence of collective dispersal. Arguments to this effect have been made before (*12*, *14*, *19*, *55*) and are supported by simulations indicating that CGP can theoretically be produced by a combination of a strong sweepstake event and mild collective dispersal (*14*). However, in our empirical case study, collective dispersal rates upward of 90% were required to generate a comparable pattern of CGP, even when assuming an extreme sweepstake event involving a single reproducing female. While this high rate of inferred collective dispersal may be unexpected, there is some evidence to suggest that marine larvae can remain in cohesive cohorts for several weeks at a time (*56*).

In the case of *N. concinna*, we envisage that a large cohort of offspring from a single stack may have hatched during a slack tide with little water movement. Their positive phototaxis after hatching (*41*) would result in the larvae coalescing toward the surface, after which they must have been moved offshore before reaching competence, thereby making a large proportion of larvae ready to settle when they reached Rose Garden. Alternatively, it is worth considering whether the larvae could have been swept into a tidepool and remained there until metamorphosis and settlement. However, this is very unlikely as there are few rockpools this far south in Antarctica, because intertidal ice scour prevents them from forming. The shoreline where these limpets were collected at Rose Garden is also steep, and there are no rockpools in the vicinity. It is furthermore unlikely that the larvae could have been swept into a high-shore rockpool, as these rarely stay liquid for more than a few days, because they freeze. Moreover, precipitation in coastal sites would likely reduce the salinity of any high-shore rockpools beyond the tolerance limit of *N. concinna* larvae. Another possibility is that, in some marine species like *C. intestinalis*, collective larval dispersal can be mediated by the retention of newly hatched larvae in mucous strings (*57*). However, Peck *et al.* (*41*) observed *N. concinna* larvae moving as individuals and actively swimming (*41*), while to our knowledge no one has ever observed such mucus-entrained larvae in Patellid limpets.

In line with the outcomes of our simulations, there was no evidence for outlier loci associated with CGP. This does not necessarily preclude a role of natural selection given the difficulty of detecting selective signals against genomic backgrounds shaped by long and often complex histories of divergence, especially for traits with polygenic architectures where signals attributable to any one locus may be relatively weak (*58*). However, several aspects of our study design are well suited to detecting genomic signatures of selection. First, genome scans are particularly powerful on microgeographic scales where the genomic background is relatively homogenous (*23*), as is the case for *N. concinna* within Ryder Bay. Second, temporally replicated genome scans should, in principle, allow spurious signals associated with shared demographic histories to be separated from genuine outlier loci influenced by selection. Third, although the genome size of *N. concinna* is unknown, related limpet species of the superfamily Lottoidea have genome sizes in the order of 0.5 Gb. Assuming a similar genome size, our marker density should be somewhere in the order of one SNP per 5 kb, which should be sufficient to detect at least some of the larger genomic regions influenced by selection, if indeed these exist. Last, although there is some evidence to suggest that selection at specific loci can contribute to fine-scale patchiness in organisms inhabiting highly heterogenous habitats such as rocky shores (*59*), especially where iceberg scour adds to the patchiness (*60*), all of our *N. concinna* samples were gathered from the subtidal zone where the environment is more stable and predictable. Consequently, post-settlement selection is unlikely to be anywhere near as strong as in intertidal habitats, again supporting our results.

Last, highly fecund populations abound in nature, in particular among broadcast spawners such as *N. concinna*. High fecundity combined with type III survivorship curves provides opportunities for sweepstakes reproduction, i.e., the random production of huge numbers of offspring from a small number of parents (*8*). An open but important question is whether such reproductive events occur often enough to have an impact on the evolution of the wider population. Classical models of genetic reproduction such as the Wright-Fisher model do not admit sweepstakes reproduction. It is therefore clear that the presence of sweepstakes reproduction would fundamentally alter our view of evolution, because nearly all of population genetics theory is based on the Wright-Fisher model and the associated Kingman coalescent. However, repeated strong bottlenecks and repeated selective sweeps are capable of generating patterns of genetic variation similar to those produced by sweepstakes reproduction, and it has been shown that both processes can produce genealogies that are better approximated by multiple-merger coalescents than by the Kingman coalescent (*61*, *62*). While further research is needed to distinguish among multiple-merger coalescent models produced by different biological processes, these mathematical results, in addition to our empirical findings, suggest that multiple-merger coalescent models are relevant for understanding genetic variation in natural populations.

Logistic constraints meant that we were unable to replicate fine-scale sampling farther afield from Ryder Bay. Thus, although our results strongly suggest that sweepstake events may be common and widespread in *N. concinna*, we would ideally like to know more about patterns of spatial and temporal variation and how these may relate to variation in the prevailing oceanographic conditions. Unfortunately, a dedicated cruise would be needed to extend our sampling northward along the Antarctic Peninsula. However, an alternative approach would be to sample exhaustively from a limited number of locations within Ryder Bay, stratify the samples by age, and then reconstruct temporal patterns by analyzing each cohort separately.

In conclusion, our study shows that unexpected patterns of fine-scale genetic differentiation can be produced by a combination of sweepstakes reproductive success and collective dispersal, in support of theoretical arguments (*7*, *8*) and simulation studies (*14*). By implication, breeding groups of marine organisms can be tiny despite very large census population sizes. This may provide a general explanation for the unexpectedly low effective population sizes of many marine species (*8*), although *N*_e_ estimates for continuously distributed species can be downwardly biased (*63*) and other factors such as life history and demographic variation will also play a role (*64*). In addition, our study provides indirect evidence for strong collective dispersal, which has previously been described as one of the most intriguing yet speculative aspects of CGP (*7*). Together, our results are important for understanding the evolutionary dynamics of marine populations and highlight the need for further studies of the evolutionary impacts of sweepstake reproduction and of the wider prevalence of collective dispersal.

## MATERIALS AND METHODS

### Sample collection

Antarctic limpets were collected by scuba divers during the austral summers of 1999 and 2015 from depths of 5 to 10 m. Samples were collected at random from each locality within an area of approximately 3 m by 3 m. At the first time point, we gathered samples from five sites along the Antarctic Peninsula as well as from four additional localities within Ryder Bay, Adelaide Island (Fig. 1). At the second time point, temporal replicates were collected from all five locations within Ryder Bay. To ensure that our sampling focused on a single age cohort, sampling was restricted to animals with shells between 20 and 30 mm long [corresponding to animals approximately 10 years of age (*65*)]. A small piece of foot tissue was excised from each limpet and stored in 96% ethanol at −20°C.

### Drifters

To evaluate general current circulation patterns in and around Ryder Bay, three Clearsat-15 surface drifters (platform IDs 101562, 101827, and 101959) were deployed from rigid inflatable boats close to Rothera Research Station, Adelaide Island. These were configured with conventional drogues at 15 m depth and returned GPS position fixes via satellite. Full-time periods of drifter data spanned from 10 January 2012 to 3 May 2012 inclusive. To focus data collection within Ryder Bay, attempts were made to recover and redeploy the drifters when they approached the edge of the boating limit around Rothera. Only data flagged as location category three (the most precise Argos data) were analyzed. Velocity thresholding (using adjacent position pairs) and manual editing were used to exclude erroneous data: These occurred during periods when the drifters were ashore at Rothera, when they had grounded close to an island, or when they were on a boat following recovery and before redeployment.

### DNA extraction and RAD sequencing

Whole genomic DNA was extracted using an adapted phenol-chloroform protocol (*66*) and sent to the Beijing Genomics Institute (BGI) for RAD sequencing (*67*). RAD libraries were prepared using Eco RI and 150-bp paired-end sequenced on an Illumina X Ten. Clean demultiplexed reads were de novo assembled into RAD loci using Stacks version 2.52 (*68*). The main parameters –M and –n were both set to 6 following the optimization procedure described by Rochette and Catchen (*69*). The resulting SNPs were quality-filtered using VCFtools (*70*) and PLINK version 1.9 (*71*). Detailed descriptions of the library preparation, sequencing, and bioinformatics pipeline are provided in Supplementary Methods.

### Data analysis

Population genetic structure was analyzed using three complementary approaches. First, we implemented sparse nonnegative matrix factorization (sNMF) using the R package LEA (*72*). Second, we computed pairwise *F*_st_ values among all populations separately for the two geographic scales and determined statistical significance with 10,000 bootstrap replicates using the R package StAMPP (*73*). The resulting *P* values were then Bonferroni-corrected for multiple tests. The *F*_st_ values were also used in Mantel tests to evaluate the significance of isolation-by-distance patterns. Third, we implemented PCA using the R package adegenet (*74*).

Pairwise genomic relatedness (PI_HAT) values, which reflect the overall proportion of the genome that is IBD, were calculated among all individuals within each population using PLINK. The output from PLINK, including *Z*0, *Z*1, and *Z*2 values, which reflect the proportion of the genome where a pair of individuals share zero, one, or two alleles IBD, was also used to assign pairs of individuals to relatedness categories following the approach of Manichaikul *et al.* (*30*). For comparison, we also inferred pairwise genomic relatedness and assigned individuals to kinship categories using the software KING (*30*). The genetic diversity of each population was estimated by computing genome-wide nucleotide diversity (π) in SambaR (*75*), and the magnitude of LD within each population was quantified by calculating *r*^2^ values between all possible pairs of loci in PLINK. *N*_e_ estimates were obtained using the LD and molecular coancestry methods within NeEstimator (*50*). Last, we searched for loci potentially under selection within Ryder Bay by conducting a genetic outlier analysis in BayPass (*47*). The –log_10_(*P* value) threshold to determine genome-wide significance was adjusted for multiple tests using Bonferroni correction. Further details are available in Supplementary Methods.

### Forward genetic simulations

Forward genetic simulations were implemented in SLiM (*31*) as described in Supplementary Methods. First, we simulated a single population where, at the start of each generation, individuals were divided into spawning aggregations or stacks of up to 20 limpets. The sex ratio within each stack was set to 1:1. We then selected a single stack and allowed a variable number of females (*F*) to reproduce at random with opposite sex individuals from the same stack. Offspring were produced until the carrying capacity (*K*) of the population was reached. We first conducted exploratory simulations to investigate the effects of varying *F*, *K*, and the number of individuals in a stack (*St*). We then sampled 10 individuals at random from the resulting offspring, assigned them to kinship categories based on the pedigree, and then defined simulations as “successful” when only full- and half-siblings were recovered. In this way, we determined the optimal combination of parameter values for *F*, *K*, and *St*.

Next, we simulated a generalized scenario comprising five populations, which included a source and a sink. We modeled reproduction at the source population using the optimal combination of parameter values for *F*, *K*, and *St* from the previous simulations. In the other four populations, multiple females within multiple stacks were allowed to reproduce. We then allowed varying proportions of offspring generated at the source population to collectively disperse to the sink population. Migration among the populations was then implemented using a stepping stone model until all five populations reached their carrying capacity. Simulations were again regarded as successful when they replicated the observed kinship structure of the Rose Garden 1999 cohort.

Last, we allowed the previous simulations to run for a further three generations without enforcing any additional sweepstake events and setting *CD* to zero. During these additional simulated generations, all populations were connected by migration according to a stepping stone model. We then extracted samples of 10 individuals from each population at each time point and calculated their nucleotide diversity and kinship structure.

### Analysis of allele frequency spectra

The allele frequency spectrum is a simple summary statistic that provides useful information about genetic variation among individuals. Let *X_j_* (*n*) denote the number of new (derived) mutations observed in *j* copies in a sample of *n* sequences. The vector (*X*_1_(*n*), …, *X*_*n*−1_(*n*)) is known as the site frequency spectrum. We worked with the folded spectrum, defined as *Y_j_*(*n*) = *X_j_*(*n*) + *X*_*n*−*j*_(*n*) for 1 ≤ *j* < *n*/2. The allele frequency spectrum is closely related to the “edge-length spectrum” (*B_i_*(*n*),...,*B*_*n*−1_(*n*)), where *B_i_*(*n*) is the random length of edges (branches) supporting *i* leaves (or DNA sequences). Under the infinitely many sites model, E[*X_i_*(*n*)] = θE [*B_i_*(*n*)], where θ is the scaled mutation rate. We defined *Z_i_*(*n*) = *X_i_*(*n*)/*X*(*n*), where *X*(*n*) = *X*_1_(*n*) + … + *X*_*n*−1_(*n*) as the random total number of segregating sites. Then, E[*Z_i_*(*n*)] is well approximated by E[*B_i_*(*n*)]/E[*B*(*n*)] (*39*). The exact normalized expected allele frequency spectrum, i.e., E[*B_i_*(*n*)]/E[*B*(*n*)], for the Xi-Beta(2-α, α) coalescent was computed using recursions (*76*) and compared to the folded normalized allele frequency spectrum of each subpopulation (cohort) using the metric *l*_2_ *=*$\sqrt{\sum_{j=1}^{n/2}{\left(x_{j}-y_{j}\right)}^{2}}$, where *x_j_* and *y_j_* are the coordinates of the spectra being compared. The Xi-Beta(2-α, α) coalescent is a simultaneous multiple-merger coalescent obtained from a population model of sweepstakes reproduction in a diploid population (*34*, *52*). A multiple-merger in the sample is then due to a single pair of diploid parents having a large number of offspring (on the order of the population size) and involving a number of the ancestral lineages of the sample, which can then simultaneously merge in up to four groups (*76*, *77*).

## Supplementary Materials

### This PDF file includes:

Supplementary Methods Figs. S1 to S4 Tables S1 to S6 References

## Notes

View/request a protocol for this paper from *Bio-protocol*.
