# Supplementary material for: Sweepstake reproductive success and collective dispersal produce chaotic genetic patchiness in a broadcast spawner
Source: Sci Adv. 2021 Sep 10;7(37):eabj4713. doi: 10.1126/sciadv.abj4713 (PMC8442859; doi:10.1126/sciadv.abj4713)
Supplement: Supplementary file 1 — Supplementary Methods Figs. S1 to S4 Tables S1 to S6 References [file sciadv.abj4713_sm.pdf]

Science Advances
AAAS

Supplementary Materials for

Sweepstake reproductive success and collective dispersal produce chaotic genetic patchiness in a broadcast spawner

David L. J. Vendrami, Lloyd S. Peck, Melody S. Clark, Bjarki Eldon,
Michael Meredith, Joseph I. Hoffman*

*Corresponding author. Email: joseph.hoffman@uni-bielefeld.de

Published 10 September 2021, *Sci. Adv.* **7**, eabj4713 (2021)
DOI: 10.1126/sciadv.abj4713

This PDF file includes:

Supplementary Methods
Figs. S1 to S4
Tables S1 to S6
References

Supplementary Methods

RAD library preparation and sequencing

Whole genomic DNA was sent to the Beijing Genomics Institute (BGI) for Restriction Site Assoociated DNA (RAD) sequencing (*67*). 1 μg of DNA from each sample was first digested with the enzyme EcoRI (New England Biolabs). A high-fidelity enzyme was used to avoid star activity. Next, Illumina P1 index adapters were ligated to the digested DNA by incubating them with a ligation reaction mix for one hour at 20° C. Afterwards, the resulting fragments were then pooled in equal volumes and randomly sheared using a Covaris sonicator. These were then run on an agarose gel and DNA fragments between 300 and 500 bp in size were excised and purified using a QIAquick PCR purification kit (Qiagen). Fragmented DNA was then incubated at 20° C for 30 minutes with end repair mix (prepared in-house) and further purified using a QIAquick PCR purification kit. This was followed by incubation at 37° C for 30 minutes with A-tailing mix (prepared in-house) and was purified using a QIAquick PCR purification kit. Illumina P2 adapters were then ligated to the adenylated 3' ends by incubation at 20° C for 15 minutes and the products were further purified with a QIAquick PCR purification kit. Afterwards, 12 PCR cycles were performed using a proprietary PCR primer cocktail (prepared in-house) and VeraSeq 2.0 master mix (Enzymatics) to enrich the adapter ligated DNA fragments. The resulting libraries were validated using a 2100 bioanalyzer (Agilent) and an ABI StepOnePlus Real-Time PCR System (Thermofisher). Finally, the qualified libraries were 150 paired-end sequenced on an Illumina Xten machine. During library preparation, the sampling cohorts were pooled at random within three libraries (Table S1), each of which was sequenced on an Illumina lane within the same Flow Cell to avoid potential batch effects.

Bioinformatic analysis

Already demultiplexed and cleaned sequence reads were obtained from BGI and further quality checked using FastQC (http://www.bioinformatics.babraham.ac.uk/projects/fastqc/). All of the samples were retained for subsequent analysis as they contained similar numbers of reads (mean = 4,769,382, sd = 3,149,063, range: 720,804–19,432,533). The sequence reads were *de novo* assembled into RAD loci using Stacks 2.52 (*68*). Values for the two main parameters –M and –n were chosen following the optimization procedure described by Rochette & Catchen (*69*). Briefly, as many of the populations in our dataset are likely to be closely related, we set –M = –n (*84*) and evaluated the performance of values ranging from one to nine in a randomly selected subset of 12 samples. The combination of these parameters for which the number of polymorphic loci present in at least 80% of individuals reached a plateau was defined as optimal. The optimal combination (–M = 6 and –n = 6) was then selected for analyzing the entire dataset. The resulting catalog of *de novo* assembled RAD loci was filtered to retain only RAD loci that were found in at least 80% of the samples, in order to avoid the risk of incorporating RAD loci erroneously assembled in a subset of samples. This threshold was based on visual inspection of the distribution of loci coverage across samples. PCR duplicates were then identified and removed by specifying the --rm-pcr-duplicates option within the gstacks module. The resulting raw SNP genotypes were then filtered to retain only biallelic SNPs with genotype quality and depth of coverage greater than five using VCFtools (*70*) as well as

to retain only SNPs genotyped in at least 90% of individuals. Subsequently, we discarded all SNPs with a depth of coverage greater than twice the mean depth of the raw dataset (> 27) in order to filter out potentially paralogous loci. Next, all individuals with more than 30% missing data were removed and only variants with MAF greater than 0.01 were retained. Finally, the software PLINK version 1.9 (*71*) was used to filter out SNPs showing significant departures from Hardy-Weinberg equilibrium (HWE) with an alpha level of 0.05 after having implemented mid-*p* adjustment (*85*) and pruned out putatively linked loci using an r^2 threshold of 0.5. The depth of coverage of the SNPs retained in the final dataset was quantified using VCFtools in order to ensure that no significant differences were present among the three sequencing libraries.

Outlier analysis

Temporally replicated genome scans for outlier loci were performed on the cohorts sampled within Ryder Bay in 1999 and 2015 using the software BayPass (*47*). This program implements a Bayesian hierarchical model approach to identify loci that are potentially under divergent selection while accounting for evolutionary relationships among the samples. To do this, BayPass estimates a covariance matrix of allele frequencies across populations, which is informative about population history and provides a comprehensive description of population structure. We ran BayPass under the core model with default parameters and specifying five different populations, corresponding to the five different sampling locations. To evaluate genome-wide statistical significance, we used a Bonferroni adjusted $-\log_{10}$ (*p*-value) threshold of 6.34. To check whether we might have inadvertently removed loci under selection from our dataset by filtering out SNPs showing significant deviations from HWE, we repeated this analysis using a dataset that was not filtered for HWE. This dataset consisted of 112,246 SNPs and the corresponding Bonferroni adjusted $-\log_{10}$ (*p*-value) threshold to determine significance was then set to 6.35.

Simulations

Forward genetic simulations were implemented in SLiM (*31*) to investigate the specific conditions under which our empirical results for the Rose Garden 1999 cohort could be replicated. In each simulation, individuals were modelled as diploid organisms with a 100 kb genome size, with neutral mutations occurring at a rate of 1×10^{-5} and the recombination rate set to the default value of 1×10^{-8}. These parameter settings were chosen to allow the simulations to be completed within a reasonable amount of time, while also allowing for a common baseline level of nucleotide diversity to establish in the simulated populations. All of the simulations were initially run for 200 generations during which all populations were connected by random migration.

Our simulations were built using a non Wright-Fisher modeling approach, which allowed us to replicate the reproductive behavior of *N. concinna*. Specifically, this species aggregates in “stacks” of up to 20 individuals prior to spawning, which is believed to maximize egg fertilization rates by enhancing spawning synchrony and the proximity of gametes (*51, 86, 87*). Consequently, at the start of each generation, we divided individuals into equal sized groups representing stacks, and only individuals within the same stack were allowed to reproduce. The sex ratio within each stack was set to 1:1 to

reflect empirical estimates of the adult sex ratio for *N. concinna* at King George Island on the Antarctic Peninsula (*88*). Opposite sex individuals within a stack were mated at random and could generate offspring with multiple partners within the same stack. Reproduction continued until the carrying capacity (*K*) of the population was reached. We modeled high variance in reproductive success according to a Weibull distribution, with scale and shape parameters of $\lambda = 0.4$ and $k = 0.55$ for non-sweepstake reproduction and $\lambda = 0.4$ and $k = 0.22$ for sweepstake reproduction. To reflect the sampling design of our empirical study, overlapping generations were modeled.

The first set of simulations attempted to evaluate the conditions that emulate the empirical kinship structure of the Rose Garden sampling cohort in 1999. We simulated a single population in which we forced a strong sweepstake event to occur by allowing only a limited number of females (*F*) located within a single stack to reproduce. We evaluated the effects of varying the number of reproducing females within the stack (*F*), the size of the stack (*St*), and *K* on the kinship structure of the resulting offspring cohort, which was subsampled to ten individuals to reflect our empirical sample size. Specifically, we tested values of *F* between 1 and 5, values of *St* equal to 5, 10 or 20, and values of *K* equal to 500, 1000 or 2000, and ran 100 simulation for each possible combination of parameters values. We then treated a simulation as having successfully replicated our empirical results when the offspring sample contained only full and half-siblings. For comparison, simulations involving multiple reproducing females (i.e. $2 \leq F \leq 5$) were re-run while allowing reproducing females to be located on different stacks.

The second set of simulations aimed at exploring the importance of collective dispersal for the generation of CGP and for the detection of sweepstake signatures. We simulated a total of five populations which included a 'source' and a 'sink' population. Reproduction at the source followed the sweepstake model described above, while using the parameter settings $F = 1$, $K = 1000$ and $St = 10$, which were found to be optimal in the first set of simulations. In the other four populations, we implemented non-sweepstake reproduction, with multiple stacks producing offspring and multiple females breeding within stacks. We then allowed varying proportions of the offspring generated at the source population to collectively disperse (*CD*) to the sink population, while all other migration among populations occurred following a unidimensional stepping stone model until carrying capacities were reached. We evaluated 12 values of *CD* (0, 10, 20, 30, 40, 50, 60, 70, 80, 90, 95 and 100) and ran 100 simulations for each value. Again, the resulting cohorts at the sink population were subsampled to ten individuals and we regarded a simulation as having successfully replicated our empirical results when the sampled offspring contained only full and half-siblings.

Finally, we selected the combination of parameters that provided the best fit to the empirical kinship structure of Rose Garden in 1999 (*F = 1, K = 1000, St = 10 and CD = 100*) and ran the simulations for three further generations without enforcing any additional sweepstake events. The aim of this analysis was to understand how the occurrence of a sweepstake event followed by collective dispersal might affect the kinship structure and genetic diversity of adjacent populations in subsequent generations. At each timepoint, the simulated cohorts were again subsampled to ten individuals, which

were used to calculate nucleotide diversity and kinship. A total of 100 simulations were run.

Fig. S1.

Genome scans for outlier loci on the microgeographic scale. Panels (a) and (b) show results for 1999 and 2015 respectively based on a Hardy-Weinberg equilibrium (HWE) filtered dataset of 109,760 SNPs. Panels (c) and (d) show results for 1999 and 2015 respectively using a dataset of 112, 246 SNPs that was not HWE filtered. Shown are Manhattan plots of minus $\log_{10}$ *P*-values for each SNP inferred from an outlier analysis implemented in BayPass (see Supplementary methods for details). The SNPs are shown in arbitrary order and colour-coded according to blocks of 10,000 SNPs. The orange dashed line represents the Bonferroni corrected genome-wide significance threshold corresponding to α = 0.05.

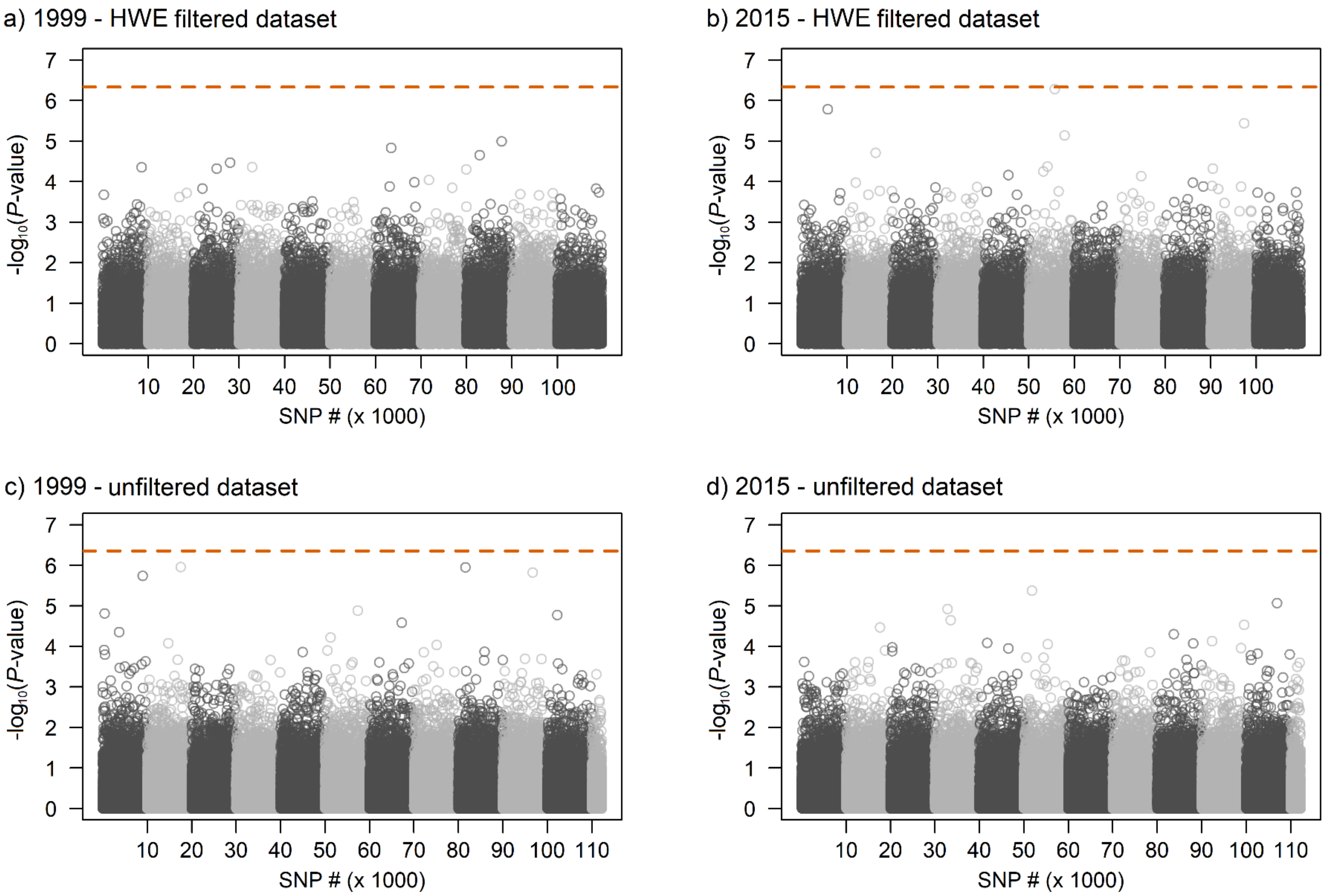

Fig. S2.

Results of exploratory simulations of sweepstake events in *N. concinna* (see Supplementary methods for details). At the start of each generation, we randomly built stacks of up to 20 individuals with a 1:1 sex ratio. We then selected a focal stack at random and allowed individuals within that stack to randomly mate until the carrying capacity (*K*) of the population was reached. We explored the effects of varying the number of reproducing females within the stack (*F*), the size of the stack (*St*), and *K* on the kinship structure of the resulting offspring cohort, which was subsampled to ten individuals to reflect our empirical sample size. A simulation was regarded as having successfully reproduced our empirical results for Rose Garden 1999 when the resulting offspring sample contained only full and half-siblings. (a) shows the proportion of successful simulations for different combinations of parameter values for *K*, *St* and *F*. (b) summarizes the kinship structure of the subsampled simulated offspring cohorts. The proportion of unrelated individuals, full siblings and half siblings are shown in orange, purple and green respectively, with points and vertical bars representing the mean and standard deviations of 100 simulations respectively.

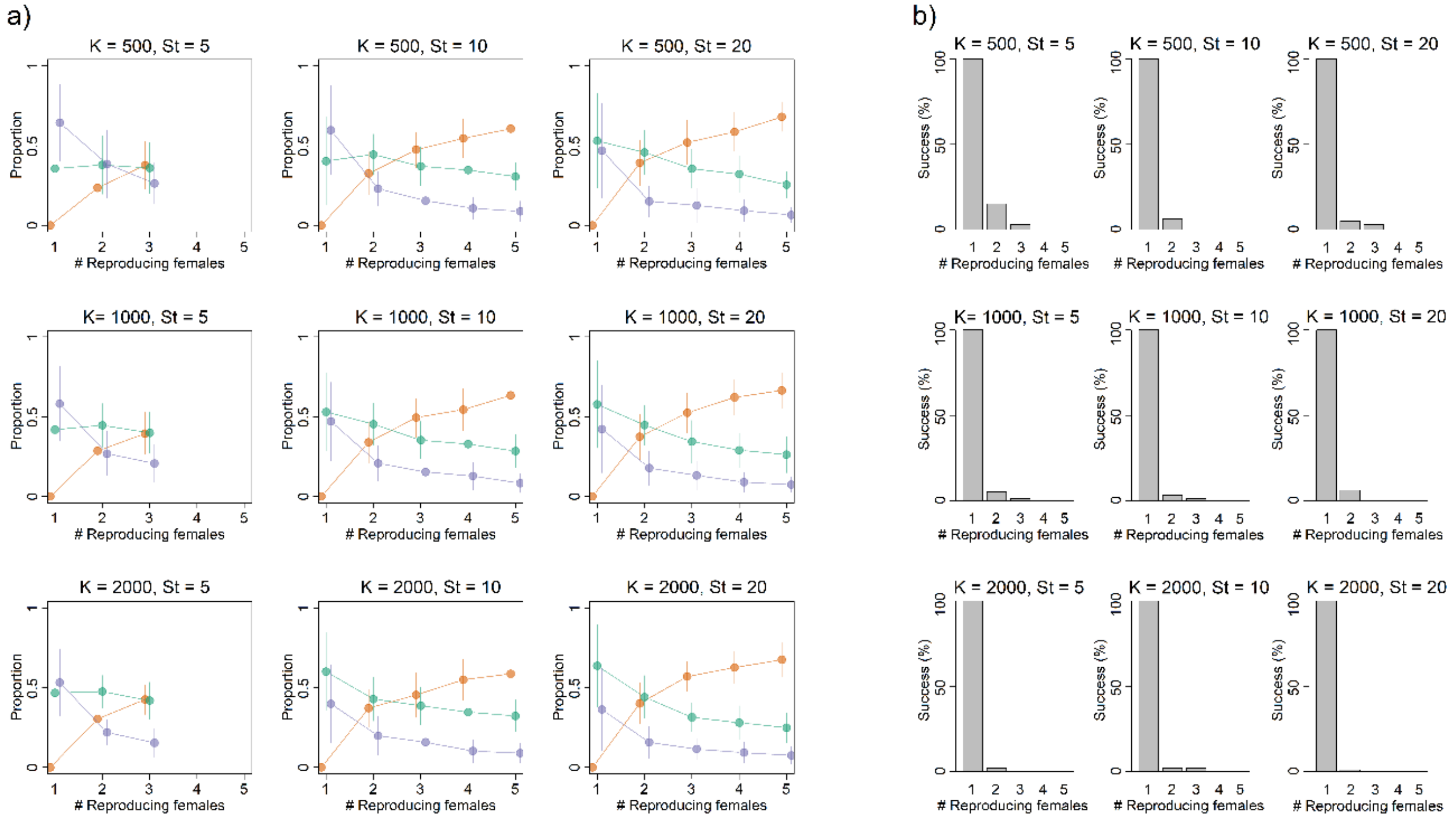

Fig. S3.

Results of exploratory simulations in which females from more than one stack were allowed to reproduce (see Supplementary methods for details). At the start of each generation, we randomly built stacks of up to 20 individuals with a 1:1 sex ratio. We then allowed between two and five females, each from a different stack, to mate at random within their stacks until the carrying capacity (*K*) of the population was reached. We explored the effects of varying the number of reproducing females (*F*), the size of the stack (*St*), and *K* on the kinship structure of the resulting offspring cohort, which was subsampled to ten individuals to reflect our empirical sample size. A simulation was regarded as having successfully reproduced our empirical results for Rose Garden 1999 when the resulting offspring sample contained only full and half-siblings. (a) shows the proportion of successful simulations for different combinations of parameter values for *K*, *St* and *F*. (b) summarizes the kinship structure of the subsampled simulated offspring cohorts. The proportion of unrelated individuals, full siblings and half siblings are shown in orange, purple and green respectively, with points and vertical bars representing the mean and standard deviations of 100 simulations respectively.

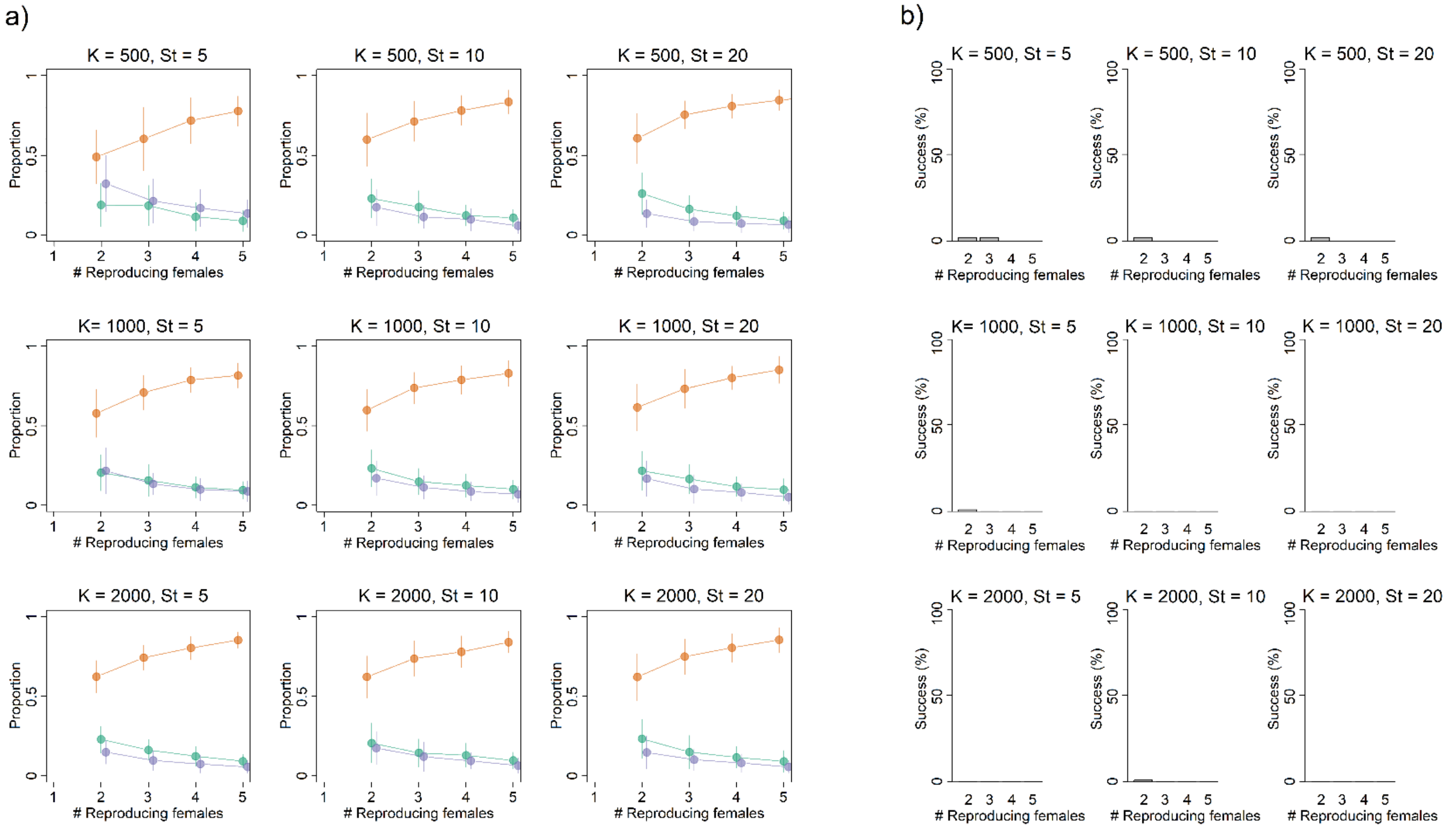

Fig. S4.
Simulated nucleotide diversity and kinship structure in the three generations following the sweepstake event (see Supplementary methods for details). We simulated a total of five populations including a 'source' (So) and a 'sink' (Si) population and allowed varying proportions of the offspring generated at the source population to collectively disperse to the sink (*CD*), while migration among the other populations (labelled 1, 2 and 3) followed a stepping stone model (see Supplementary methods for details). Simulations then proceeded for three further generations where no further sweepstake events were enforced and migration among all five populations followed a stepping stone model. The boxplot shows variation in nucleotide diversity across cohorts sampled from the five simulated populations at a) one generation after the original sweepstake event; b) two generations afterwards; and c) three generations afterwards. Nucleotide diversity is expressed as the proportion of the maximum value observed within each simulation, with the sink population highlighted in purple. The barplots show proportions of unrelated individuals (orange), half-siblings (green) and full-siblings (purple) in cohorts sampled from the five simulated populations.

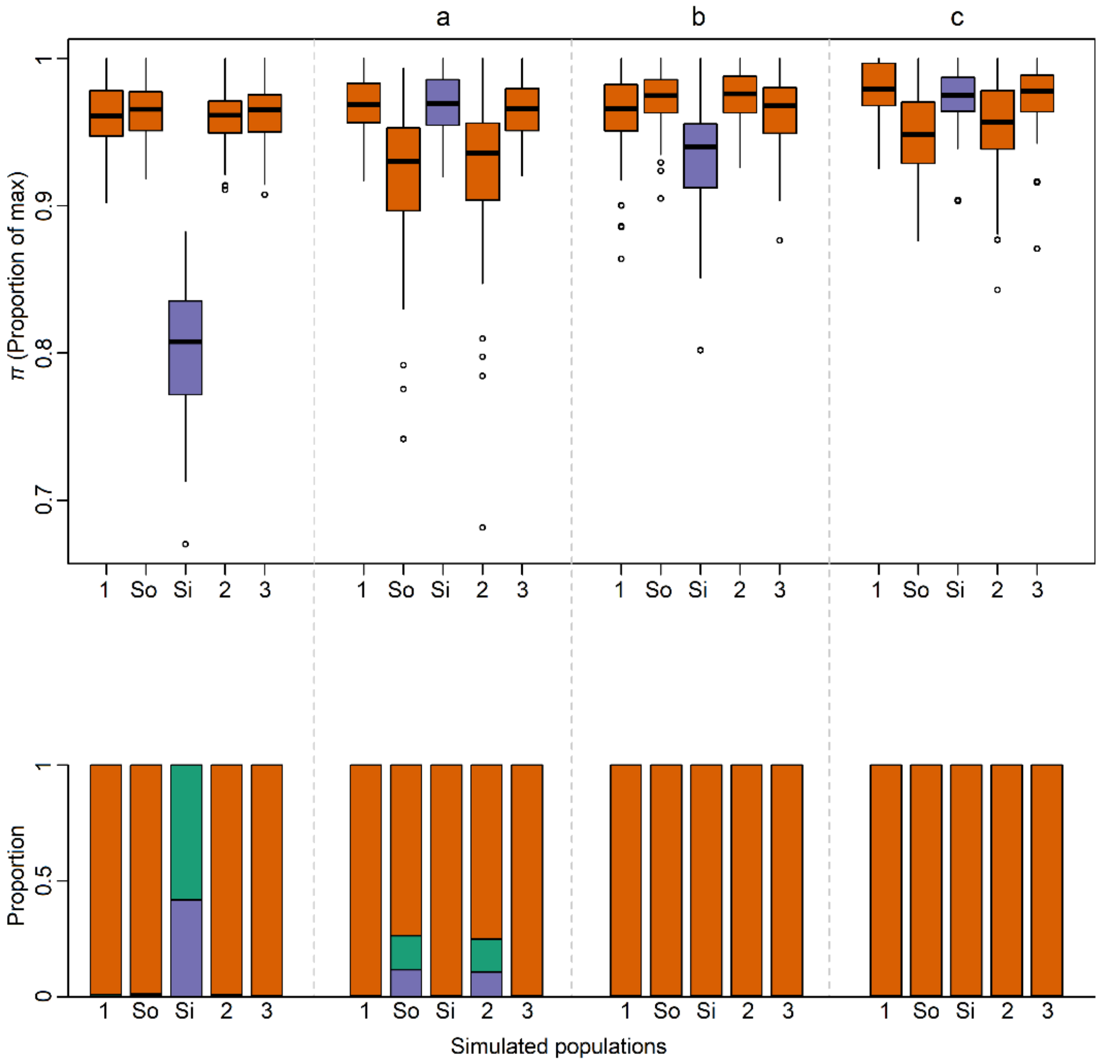

Table S1.

Pooling strategy during the library preparation and related summary statistics. The sampling cohorts were assigned at random to one of three sequencing libraries as shown in the table. To provide an indication of the quality of the sequencing data obtained from each library, we calculated the mean (± standard deviation) per-base phred-scaled quality scores and the mean (± standard deviation) depth of coverage of the SNPs retained for analysis.

Library ID	Included sampling cohorts	Average per-base quality (± SD)	Average SNP depth of coverage (± SD)
Library 1	Rose Garden 1999, Galindez 1999, Snow 1999, Signy 1999, Leonie 1999	37.82 (0.09)	12.96 (2.27)
Library 2	Dobrowolski 1999, Anchorage North 1999, Trolval 1999, Trolval 2015, East Beach 1999	37.94 (0.25)	13.29 (3.03)
Library 3	Rose Garden 2015, Anchorage North 2015, East Beach 2015, Leonie 2015	37.79 (0.08)	12.13 (4.16)

Table S2.

Pairwise F_{st} values at different geographic scales. Pairwise F_{st} values calculated among *N. concinna* cohorts sampled on (a) the macrogeographic scale; (b) the microgeographic scale in 1999; and (c) the microgeographic scale in 2015 (c). F_{st} values are given below the diagonal and Bonferroni corrected *p*-values are given above the diagonal. Significant F_{st} values after Bonferroni correction are highlighted in bold.

a) Macrogeographic scale

	Rose Garden	Galindez	Dobrowolski	Snow	Signy
Rose Garden	*	<0.01	<0.01	<0.01	<0.01
Galindez	**0.0048**	*	0.093	<0.01	<0.01
Dobrowolski	**0.0024**	0.0008	*	1	<0.01
Snow	**0.0032**	**0.0010**	-0.0004	*	<0.01
Signy	**0.0231**	**0.0221**	**0.0216**	**0.0206**	*

b) Microgeographic scale 1999

	Rose Garden	Anchorage North	Trolval	East Beach	Leonie
Rose Garden	*	<0.01	<0.01	<0.01	<0.01
Anchorage North	**0.0032**	*	0.134	<0.01	1
Trolval	**0.0035**	0.0007	*	1	1
East Beach	**0.0036**	**0.0010**	-0.0007	*	1
Leonie	**0.0042**	-0.0007	< 0.0001	0.0003	*

c) Microgeographic scale 2015

	Rose Garden	Anchorage North	Trolval	East Beach	Leonie
Rose Garden	*	1	1	1	1
Anchorage North	-0.0011	*	1	1	1
Trolval	-0.0005	-0.0001	*	1	1
East Beach	-0.0006	-0.0002	-0.0010	*	1
Leonie	-0.0007	-0.0005	-0.0003	-0.0002	*

Table S3.

Pairwise F_{st} values calculated for both geographic scales and time points combined. F_{st} values are given below the diagonal and Bonferroni corrected *p*-values are given above the diagonal. Significant F_{st} values after Bonferroni correction are highlighted in bold. SI = Signy Island, SN = Snow Island, DO = Dobrowolski Island, GA = Galindez Island, RG = Rose Garden, TR = Trolval, AN = Anchorage North, LE = Leonie North East, EB = East Beach. Cohorts from 1999 and 2015 are denoted with the suffixes '99' and '15' respectively.

	RG99	GA	DO	SN	SI	RG15	AN99	AN15	TR99	TR15	EB99	EB15	LE99	LE15
RG99	*	<0.01	<0.01	<0.01	<0.01	<0.01	<0.01	<0.01	<0.01	<0.01	<0.01	<0.01	<0.01	<0.01
GA	**0.0048**	*	<0.01	<0.01	<0.01	<0.01	<0.01	0.0377	<0.01	<0.01	<0.01	0.0667	<0.01	<0.01
DO	**0.0024**	0.0008	*	0.9238	<0.01	0.9022	0.1374	0.961	0.9054	0.9261	0.6341	0.9663	0.9986	0.0455
SN	**0.0032**	0.0010	-0.0004	*	<0.01	0.0459	0.0186	0.5542	0.4453	0.9478	0.0586	0.3375	0.2956	0.0334
SI	**0.0231**	**0.0221**	**0.0216**	**0.0206**	*	<0.01	<0.01	<0.01	<0.01	<0.01	<0.01	<0.01	<0.01	<0.01
RG15	**0.0024**	**0.0013**	-0.0004	0.0005	**0.0216**	*	0.9135	0.9998	0.5082	0.9172	0.0361	0.9719	0.382	0.9845
AN99	**0.0032**	**0.0021**	0.0003	0.0006	**0.0215**	-0.0004	*	<0.01	<0.01	0.412	<0.01	0.7424	0.9868	0.8051
AN15	**0.0036**	0.0006	-0.0006	0.0000	**0.0216**	-0.0011	**0.0010**	*	0.0331	0.6226	0.7819	0.695	0.9447	0.946
TR99	**0.0035**	**0.0012**	-0.0004	0.0000	**0.0212**	0.0000	0.0007	0.0006	*	0.9991	0.9845	0.8908	0.5103	0.9576
TR15	**0.0060**	**0.0015**	-0.0005	-0.0006	**0.0206**	-0.0005	0.0001	-0.0001	-0.0012	*	0.382	0.9962	0.9969	0.8096
EB99	**0.0036**	**0.0009**	-0.0001	0.0005	**0.0223**	0.0005	0.0010	-0.0002	-0.0007	0.0001	*	0.7713	0.1413	0.0166
EB15	**0.0028**	0.0005	-0.0006	0.0001	**0.0198**	-0.0006	-0.0002	-0.0002	-0.0004	-0.0010	-0.0002	*	0.9998	0.7492
LE99	**0.0042**	**0.0013**	-0.0009	0.0002	**0.0224**	0.0001	-0.0007	-0.0005	0.0000	-0.0010	0.0003	-0.0011	*	0.4963
LE15	**0.0031**	**0.0015**	0.0005	0.0006	**0.0202**	-0.0007	-0.0003	-0.0005	-0.0005	-0.0003	0.0007	-0.0002	0.0000	*

Table S4.

Cohort-specific linkage disequilibrium. Genome-wide pairwise linkage disequilibrium (LD) statistics are provided for each sampling cohort.

Population	Year	Mean	Standard deviation	First quartile	Median	Third quartile
Rose Garden	1999	0.1908	0.2168	0.1111	0.0278	0.2917
Galindez	1999	0.1364	0.1607	0.0735	0.0162	0.2000
Dobrowolski	1999	0.1205	0.1449	0.0625	0.0150	0.1734
Snow	1999	0.1194	0.1437	0.0625	0.0150	0.1724
Signy	1999	0.1232	0.1478	0.0645	0.0152	0.1779
Rose Garden	2015	0.1239	0.1486	0.0646	0.0154	0.1800
Anchorage North	1999	0.1184	0.1428	0.0625	0.0148	0.1667
Anchorage North	2015	0.1472	0.1730	0.0832	0.0192	0.2143
Trolval	1999	0.1226	0.1473	0.0645	0.0152	0.1776
Trolval	2015	0.2169	0.2331	0.1385	0.0294	0.3333
East Beach	1999	0.1163	0.1404	0.0625	0.0145	0.1667
East Beach	2015	0.1434	0.1677	0.0801	0.0182	0.2105
Leonie	1999	0.1179	0.1423	0.0625	0.0148	0.1667
Leonie	2015	0.1337	0.1579	0.0714	0.0161	0.1957

Table S5.

Cohort-specific effective population size. Effective population size estimates were calculated for each cohort using the linkage disequilibrium (N_{eLD}) and the molecular coancestry (N_{eC}) methods implemented in $N_e$estimator (*50*). 95% confidence intervals are reported in parentheses.

Population	Year	N_{eLD}	N_{eC}
Rose Garden	1999	NA	2.5 (2.4–2.5)
Galindez	1999	155.4 (154.8–156)	5.0 (4.8–5.1)
Dobrowolski	1999	151.9 (151–152.8)	4.9 (4.8–5.1)
Snow	1999	210.5 (209.8–211.2)	11.2 (10.8–11.6)
Signy	1999	295.2 (294.6–295.8)	11.7 (11.1–12.4)
Rose Garden	2015	188.4 (187.9–188.9)	5.9 (5.8–5.4)
Anchorage North	1999	247.9 (247.3–248.5)	5.6 (5.4–5.8)
Anchorage North	2015	306.2 (305–307.4)	2.4 (2.4–2.5)
Trolval	1999	166.4 (165.8–167)	3.4 (3.3–3.5)
Trolval	2015	NA	4.9 (4.7–5.0)
East Beach	1999	227.8 (227.3–228.3)	7.5 (7.3–7.8)
East Beach	2015	236.1 (235.5–236.7)	5.3 (5.2–5.4)
Leonie	1999	475.4 (474.8–476.1)	4.5 (4.4–4.7)
Leonie	2015	106.6 (106.1–107.1)	7.1 (6.9–7.3)

Table S6.

Goodness of fit between observed and expected allele frequency spectra for each cohort using the l_2 metric. l_2 (Xi-Beta) refers to distances to allele frequency spectra expected under the Xi-Beta (2–α, α) coalescent model, while l_2 (Kingman) refers to distances to allele frequency spectra expected under the Kingman coalescent model.

Population	Year	α	l_2 (Xi-Beta)	l_2 (Kingman)
Rose Garden	1999	1.42	0.074	0.105
Galindez	1999	1.40	0.051	0.087
Dobrowolski	1999	1.44	0.052	0.087
Snow	1999	1.45	0.051	0.086
Signy	1999	1.47	0.053	0.085
Rose Garden	2015	1.46	0.053	0.087
Anchorage North	1999	1.45	0.05	0.086
Anchorage North	2015	1.48	0.067	0.094
Trolval	1999	1.43	0.053	0.09
Trolval	2015	1.16	0.068	0.109
East Beach	1999	1.42	0.047	0.087
East Beach	2015	1.37	0.057	0.094
Leonie	1999	1.47	0.055	0.086
Leonie	2015	1.39	0.052	0.089

REFERENCES AND NOTES

1. J. P. Kritzer, P. F. Sale, Metapopulation ecology in the sea: From Levins' model to marine ecology and fisheries science. *Fish Fish.* **5**, 131–140 (2004).
2. A. C. Stier, A. M. Hein, V. Parravicini, M. Kulbicki, Larval dispersal drives trophic structure across Pacific coral reefs. *Nat. Commun.* **5**, 5575 (2014).
3. K. Selkoe, C. C. D'Aloia, E. D. Crandall, M. Iacchei, L. Liggins, J. B. Puritz, S. von der Heyden, R. J. Toonen, A decade of seascape genetics: Contributions to basic and applied marine connectivity. *Mar. Ecol. Prog. Ser.* **554**, 1–19 (2016).
4. S. R. Palumbi, Population genetics, demographic connectivity, and the design of marine reserves. *Ecol. Appl.* **13**, S146–S158 (2003).
5. L. W. Botsford, A. Hastings, S. D. Gaines, Dependence of sustainability on the configuration of marine reserves and larval dispersal distance. *Ecol. Lett.* **4**, 144–150 (2001).
6. L. Hauser, G. R. Carvalho, Paradigm shifts in marine fisheries genetics: Ugly hypotheses slain by beautiful facts. *Fish Fish.* **9**, 333–362 (2008).
7. B. Eldon, F. Riquet, J. Yearsley, D. Jollivet, T. Broquet, Current hypotheses to explain genetic chaos under the sea. *Curr. Zool.* **62**, 551–566 (2016).
8. D. Hedgecock, A. I. Pudovkin, Sweepstakes reproductive success in highly fecund marine fish and shellfish: A review and commentary. *Bull. Marine Sci.* **87**, 971–1002 (2011).
9. L. V. Plough, Genetic load in marine animals: A review. *Curr. Zool.* **62**, 567–579 (2016).
10. N. Bierne, F. Bonhomme, S. Arnaud-Haond, Dedicated population genomics for the silent world: The specific questions of marine population genetics. *Curr. Zool.* **62**, 545–550 (2016).
11. M. S. Johnson, R. Black, Chaotic genetic patchiness in an intertidal limpet, *Siphonaria* sp. *Mar. Biol.* **70**, 157–164 (1982).
12. P. David, M.-A. Perdieu, A.-F. Pernot, P. Jarne, Fine-grained spatial and temporal population genetic structure in the marine bivalve *Spisula ovalis*. *Evolution* **51**, 1318–1322 (1997).
13. N. Bierne, I. Beuzart, V. Vonau, F. Bonhomme, E. Bédier, Microsatellite-associated heterosis in hatchery-propagated stocks of the shrimp *Penaeus stylirostris*. *Aquaculture* **184**, 203–219 (2000).
14. T. Broquet, F. Viard, J. M. Yearsley, Genetic drift and collective dispersal can result in chaotic genetic patchiness. *Evolution* **67**, 1660–1675 (2013).
15. K. A. Selkoe, S. D. Gaines, J. E. Caselle, R. R. Warner, Current shifts and kin aggregation explain genetic patchiness in fish recruits. *Evolution* **87**, 3082–3094 (2006).

16. J. Hogan, R. Thiessen, D. Heath, Variability in connectivity indicated by chaotic genetic patchiness within and among populations of a marine fish. *Mar. Ecol. Prog. Ser.* **417**, 263–275 (2010).
17. M. Iacchei, T. Ben-Horin, K. A. Selkoe, C. E. Bird, F. J. García-Rodríguez, R. J. Toonen, Combined analyses of kinship and Fst suggest potential drivers of chaotic genetic patchiness in high gene-flow populations. *Mol. Ecol.* **22**, 3476–3494 (2013).
18. M. R. Christie, D. W. Johnson, C. D. Stallings, M. A. Hixon, Self-recruitment and sweepstakes reproduction amid extensive gene flow in a coral-reef fish. *Mol. Ecol.* **19**, 1042–1057 (2010).
19. J. M. Yearsley, F. Viard, T. Broquet, The effect of collective dispersal on the genetic structure of a subdivided population. *Evolution* **67**, 1649–1659 (2013).
20. A. Iannucci, S. Cannicci, I. Caliani, M. Baratti, C. Pretti, S. Fratini, Investigation of mechanisms underlying chaotic genetic patchiness in the intertidal marbled crab *Pachygrapsus marmoratus* (Brachyura: Grapsidae) across the Ligurian Sea. *BMC Evol. Biol.* **20**, 108 (2020).
21. M. S. Johnson, R. Black, Pattern beneath the chaos: The effect of recruitment on genetic patchiness in an intertidal limpet. *Evolution* **38**, 1371–1383 (1984).
22. K. Johannesson, B. Johannesson, U. Lundgren, Strong natural selection causes microscale allozyme variation in a marine snail. *Proc. Natl. Acad. Sci.* **92**, 2602–2606 (1995).
23. P.-A. Gagnaire, O. E. Gaggiotti, Detecting polygenic selection in marine populations by combining population genomics and quantitative genetics approaches. *Curr. Zool.* **62**, 603–616 (2016).
24. J. B. Puritz, J. R. Gold, D. S. Portnoy, Fine-scale partitioning of genomic variation among recruits in an exploited fishery: Causes and consequences. *Sci. Rep.* **6**, 36095 (2016).
25. K. J. Miller, B. T. Maynard, C. N. Mundy, Genetic diversity and gene flow in collapsed and healthy abalone fisheries. *Mol. Ecol.* **18**, 200–211 (2009).
26. D. Veliz, P. Duchesne, E. Bourget, L. Bernatchez, Genetic evidence for kin aggregation in the intertidal acorn barnacle (*Semibalanus balanoides*). *Mol. Ecol.* **15**, 4193–4202 (2006).
27. R. S. Waples, M. Yokota, Temporal estimates of effective population size in species with overlapping generations. *Genetics* **175**, 219–233 (2007).
28. J. W. Davey, M. L. Blaxter, RADSeq: Next-generation population genetics. *Brief. Funct. Genomics* **9**, 416–423 (2011).
29. D. L. Vendrami, L. Telesca, H. Weigand, M. Weiss, K. Fawcett, K. Lehman, M S Clark, F. Leese, C. M. Minn, H. Moore, J. I. Hoffman, RAD sequencing resolves fine-scale population structure in a

benthic invertebrate: Implications for understanding phenotypic plasticity. *R. Soc. Open Sci.* **4**, 160548 (2017).
30. A. Manichaikul, J. C. Mychaleckyj, S. S. Rich, K. Daly, M. Sale, W. M. Chen, Robust relationship inference in genome-wide association studies. *Bioinformatics* **26**, 2867–2873 (2010).
31. B. C. Haller, P. W. Messer, SLiM 3: Forward genetic simulations beyond the Wright–Fisher model. *Mol. Biol. Evol.* **36**, 632–637 (2019).
32. P. Donnelly, T. G. Kurtz, Particle representations for measure-valued population models. *Ann. Probab* **27**, 166–205 (1999).
33. J. Pitman, Coalescents with multiple collisions. *Ann. Probab.* **27**, 1870–1902 (1999).
34. S. Sagitov, The general coalescent with asynchronous mergers of ancestral lines. *J. Appl. Probab.* **36**, 1116–1125 (1999).
35. J. F. C. Kingman, The coalescent. *Stoch. Process. Their Appl.* **13**, 235–248 (1982).
36. J. F. C. Kingman, Exchangeability and evolution of large populations, in *Exchangeability in Probability and Statistics,* G. Koch, F. Spizzichino, Eds. (North-Holland, Amsterdam, 1982), pp. 97–112.
37. J. F. C. Kingman, On the genealogy of large populations. *J. Appl. Probab.* **19**, 27–43 (1982).
38. B. Eldon, J. Wakeley, Coalescent processes when the distribution of offspring number among individuals is highly skewed. *Genetics* **172**, 2621–2633 (2006).
39. B. Eldon, M. Birkner, J. Blath, F. Freund, Can the site-frequency spectrum distinguish exponential population growth from multiple-merger coalescents? *Genetics* **199**, 841–856 (2015).
40. B. Eldon, Evolutionary genomics of high fecundity. *Annu. Rev. Genet.* **54**, 213–236 (2020).
41. L. S. Peck, S. Heiser, M. S. Clark, Very slow embryonic and larval development in the Antarctic limpet *Nacella polaris*. *Polar Biol.* **39**, 2273–2280 (2016).
42. D. A. Bowden, A. Clarke, L. S. Peck, D. K. A. Barnes, Antarctic sessile marine benthos: Colonisation and growth on artificial substrata over three years. *Mar. Ecol. Prog. Ser.* **316**, 1–16 (2006).
43. D. A. Bowden, A. Clarke, L. S. Peck, Seasonal variation in the diversity and abundance of pelagic larvae of Antarctic marine invertebrates. *Mar. Biol.* **156**, 2033–2047 (2009).
44. C. A. González-Wevar, T. Saucède, S. A. Morley, S. L. Chown, E. Poulin, Extinction and recolonization of maritime Antarctica in the limpet *Nacella concinna* (Strebel, 1908) during the last

glacial cycle: Toward a model of Quaternary biogeography in shallow Antarctic invertebrates. *Mol. Ecol.* **22**, 5221–5236 (2013).

45. J. I. Hoffman, L. S. Peck, K. Linse, A. Clarke, Strong population genetic structure in a broadcast-spawning Antarctic marine invertebrate. *J. Hered.* **102**, 55–66 (2011).
46. J. I. Hoffman, A. Clarke, M. S. Clark, P. Fretwell, L. S. Peck, Unexpected fine-scale population structure in a broadcast-spawning Antarctic marine mollusc. *PLOS ONE* **7**, e32415 (2012).
47. M. Gautier, Genome-wide scan for adaptive divergence and association with population-specific covariates. *Genetics* **201**, 1555–1579 (2015).
48. B. Eldon, J. Wakeley, Linkage disequilibrium under skewed offspring distribution among individuals in a population. *Genetics* **178**, 1517–1532 (2008).
49. M. Birkner, J. Blath, B. Eldon, An ancestral recombination graph for diploid populations with skewed offspring distribution. *Genetics* **193**, 255–290 (2013).
50. C. Do, R. S. Waples, D. Peel, G. M. Macbeth, B. J. Tillett, J. R. Ovenden, NeEstimatorv2: Re-implementation of software for the estimation of contemporary effective population size (*N*e) from genetic data. *Mol. Ecol. Resour.* **14**, 209–214 (2014).
51. G. B. Picken, D. Allan, Unique spawning behaviour by the Antarctic limpet *Nacella* (*Patinigera*) *concinna* (Strebel, 1908). *J. Exp. Mar. Biol. Ecol.* **71**, 283–287 (1983).
52. J. Schweinsberg, Coalescents with simultaneous multiple collisions. *Electron. J. Probab.* **5**, 1–50 (2000).
53. P. M. Buston, C. Fauvelot, M. Y. L. Wong, S. Planes, Genetic relatedness in groups of the humbug damselfish *Dascyllus aruanus*: Small, similar-sized individuals may be close kin. *Mol. Ecol.* **18**, 4707–4715 (2009).
54. J. D. Selwyn, J. D. Hogan, A. M. Downey-Wall, L. M. Gurski, D. S. Portnoy, D. D. Heath, Kin-aggregations explain chaotic genetic patchiness, a commonly observed genetic pattern, in a marine fish. *PLOS ONE* **11**, e0153381 (2016).
55. D. Hedgecock, Does variance in reproductive success limit effective population sizes of marine organisms? in *Genetics and Evolution of Aquatic Organisms,* A. Beaumont, Ed. (Chapman and Hall, London, 1994), pp. 1222–1344.
56. O. Ben-Tzvi, A. Abelson, S. D. Gaines, G. Bernardi, R. Beldade, M. S. Sheehy, G. L. Paradis, M. Kiflawi, Evidence for cohesive dispersal in the sea. *PLOS ONE* **7**, e42672 (2012).

57. I. Svane, J. N. Havenhand, Spawning and Dispersal in *Ciona intestinalis* (L.). *Mar. Ecol.* **14**, 53–66 (1993).
58. P. A. Gagnaire, T. Broquet, D. Aurelle, F. Viard, A. Souissi, F. Bonhomme, S. Arnaud-Haond, N. Bierne, Using neutral, selected, and hitchhiker loci to assess connectivity of marine populations in the genomic era. *Evol. Appl.* **8**, 769–786 (2015).
59. K. Gérard, C. Roby, N. Bierne, P. Borsa, J. P. Féral, A. Chenuil, Does natural selection explain the fine scale genetic structure at the nuclear exonGlu-5′ in blue mussels from Kerguelen? *Ecol. Evol.* **5**, 1456–1473 (2015).
60. K. M. Brown, K. P. P. Fraser, D. K. A. Barnes, L. S. Peck, Links between the structure of an Antarctic shallow-water community and ice-scour frequency. *Oecologia* **141**, 121–129 (2004).
61. M. Birkner, J. Blath, M. Möhle, M. Steinrücken, J. Tams, A modified lookdown construction for the Xi-Fleming-Viot process with mutation and populations with recurrent bottlenecks. *Alea* **6**, 25–61 (2009).
62. R. Durrett, J. Schweinsberg, A coalescent model for the effect of advantageous mutations on the genealogy of a population. *Stoch. Process. Their Appl.* **115**, 1628–1657 (2005).
63. M. C. Neel, K. McKelvey, N. Ryman, M. W. Lloyd, R. Short Bull, F. W. Allendorf, M. K. Schwartz, R. S. Waples, Estimation of effective population size in continuously distributed populations: There goes the neighborhood. *Heredity* **111**, 189–199 (2013).
64. B. E. Rieman, F. W. Allendorf, Effective population size and genetic conservation criteria for bull trout. *North Am. J. Fish. Manag.* **21**, 756–764 (2001).
65. A. Clarke, E. Prothero-Thomas, J. Beaumont, A. Chapman, T. Brey, Growth in the limpet *Nacella concinna* from contrasting sites in Antarctica. *Polar Biol.* **28**, 62–71 (2004).
66. J. Sambrook, E. F. Fritsch, T. Maniatis, *Molecular Cloning: A Laboratory Manual* (Cold Spring Harbour Laboratory Press, New York, ed. 2, 1989).
67. N. A. Baird, P. D. Etter, T. S. Atwood, M. C. Currey, A. L. Shiver, Z. A. Lewis, E. U. Selker, W. A. Cresko, E. A. Johnson, Rapid SNP discovery and genetic mapping using sequenced RAD markers. *PLOS ONE* **3**, e3376 (2008).
68. N. C. Rochette, A. G. Rivera-Colón, J. M. Catchen, Stacks 2: Analytical methods for paired-end sequencing improve RADseq-based population genomics. *Mol. Ecol.* **28**, 4737–4754 (2019).
69. N. C. Rochette, J. M. Catchen, Deriving genotypes from RAD-seq short-read data using Stacks. *Nat. Protoc.* **12**, 2640–2659 (2017).

70. P. Danecek, A. Auton, G. Abecasis, C. A. Albers, E. Banks, M. A. DePristo, R. E. Handsaker, G. Lunter, G. T. Marth, S. T. Sherry, G. McVean, R. Durbin; 1000 Genomes Project Analysis Group, The variant call format and VCFtools. *Bioinformatics* **27**, 2156–2158 (2011).
71. S. Purcell, B. Neale, K. Todd-Brown, L. Thomas, M. A. R. Ferreira, D. Bender, J. Maller, P. Sklar, P. I. W. de Bakker, M. J. Daly, P. C. Sham, PLINK: A tool set for whole-genome association and population-based linkage analyses. *Am. J. Hum. Genet.* **81**, 559–575 (2007).
72. E. Frichot, O. François, LEA: An R package for landscape and ecological association studies. *Meth. Ecol. Evol.* **6**, 925–929 (2015).
73. L. W. Pembleton, N. O. I. Cogan, J. W. Forster, StAMPP: An R package for calculation of genetic differentiation and structure of mixed-ploidy level populations. *Mol. Ecol. Resour.* **13**, 946–952 (2013).
74. T. Jombart, *adegenet*: A R package for the multivariate analysis of genetic markers. *Bioinformatics* **24**, 1403–1405 (2008).
75. M. J. de Jong, J. F. de Jong, A. R. Hoelzel, A. Janke, SambaR: An R package for fast, easy and reproducible population-genetic analyses of biallelic SNP data sets. *Mol. Ecol. Resour.* **21**, 1369–1379 (2021).
76. J. Blath, M. C. Cronjäger, B. Eldon, M. Hammer, The site-frequency spectrum associated with Ξ-coalescents. *Theor. Popul. Biol.* **110**, 36–50 (2016).
77. M. Möhle, S. Sagitov, Coalescent patterns in diploid exchangeable population models. *J. Math. Biol.* **47**, 337–352 (2003).
78. GEBCO Bathymetric Compilation Group, *The GEBCO_2019 Grid* (British Oceanographic Data Centre, National Oceanography Centre, NERC, UK, 2019).
79. I. M. Howat, C. Porter, B. E. Smith, M.-J. Noh, P. Morin, The reference elevation model of Antarctica. *Cryosphere* **13**, 665–674 (2019).
80. J. W. Thomas, A. F. R. Cooper, The SCAR Antarctic digital topographic database. *Antarct. Sci.* **5**, 239–244 (1993).
81. C. Moffat, R. C. Beardsley, B. Owens, N. P. M. van Lipzig, A first description of the Antarctic Peninsula Coastal Current. *Deep-Sea Res. II Top. Stud. Oceanogr.* **55**, 277–293 (2008).
82. D. K. Savidge, J. A. Amft, Circulation on the West Antarctic Peninsula derived from 6 years of shipboard ADCP transects. *Deep-Sea Res. I Oceanogr. Res.* **56**, 1633–1655 (2009).

83. A. F. Thompson, K. J. Heywood, S. E. Thorpe, A. H. H. Renner, A. Trasvina, Surface circulation at the tip of the Antarctic Peninsula from drifters. *J. Phys. Ocenogr.* **39**, 3–26 (2009).
84. J. Paris, J. R. Stevens, J. M. Catchen, Lost in parameter space: A road map for stacks. *Meth. Ecol. Evol.* **8**, 1360–1373 (2017).
85. J. Graffelman, V. Moreno, The mid p-value in exact tests for Hardy-Weinberg equilibrium. *Stat. Appl. Genet. Mol. Biol.* **12**, 433–448 (2013).
86. G. B. Picken, The distribution, growth, and reproduction of the Antarctic Limpet *Nacella* (*Patinigera*) *concinna* (Strebel, 1908). *J. Exp. Mar. Biol. Ecol.* **42**, 71–85 (1980).
87. D. Stanwell-Smith, A. Clarke, The timing of reproduction in the Antarctic limpet *Nacella concinna* (srebel, 1908) (Patellidae) at Signy Island, in relation to environmental variables. *J. Moll. Stud.* **64**, 123–127 (1998).
88. D. Kim, Seasonality of marine algae and grazers of an Antarctic rocky intertidal, with emphasis on the role of the limpet *Nacella concinna* Strebel (Gastropoda: Patellidae). *Ber. Polarforsch. Meeresforsch.* **397**, 120 (2001).
